# Long-term changes in northern large-herbivore communities reveal differential rewilding rates in space and time

**DOI:** 10.1371/journal.pone.0217166

**Published:** 2019-05-21

**Authors:** James D. M. Speed, Gunnar Austrheim, Anders Lorentzen Kolstad, Erling J. Solberg

**Affiliations:** 1 Department of Natural History, NTNU University Museum, Norwegian University of Science and Technology, Trondheim, Norway; 2 Norwegian Institute for Nature Research (NINA), Trondheim, Norway; Universitat Autonoma de Barcelona, SPAIN

## Abstract

Herbivores have important impacts on ecological and ecosystem dynamics. Population density and species composition are both important determinants of these impacts. Large herbivore communities are shifting in many parts of the world driven by changes in livestock management and exploitation of wild populations. In this study, we analyse changes in large herbivore community structure over 66 years in Norway, with a focus on the contribution of wildlife and livestock. We calculate metabolic biomass of all large-herbivore species across the whole region between 1949 and 2015. Temporal and spatial patterns in herbivore community change are investigated and we test hypotheses that changes in wildlife biomass are driven by competition with livestock. We find that total herbivore biomass decreased from 1949 to a minimum in 1969 due to decreases in livestock biomass. Increasing wild herbivore populations lead to an increase in total herbivore biomass by 2009. Herbivore communities have thus reverted from a livestock dominated state in 1949 (2% of large herbivore metabolic biomass comprised of wildlife species) to a state with roughly equal wildlife and livestock (48% of metabolic biomass comprised of wildlife species). Declines in livestock biomass were a modest predictor of wildlife increases, suggesting that competition with livestock has not been a major limiting factor of wild herbivore populations over the past decades. Instead there was strong geographic variation in herbivore community change, with milder lowland regions becoming more dominated by wild species, but colder mountain and northern regions remaining dominated by livestock. Our findings indicate that there has been notable rewilding of herbivore communities and herbivore-ecosystem interactions in Norway, particularly in milder lowland regions. However, Norwegian herbivores remain mostly regulated by management, and our findings call for integrated management of wild and domestic herbivores.

## Introduction

Large herbivores drive ecological and ecosystem dynamics in many terrestrial ecosystems [1–3]. Individual herbivore species have unique effects on their habitat, depending largely on species characteristics such as body and group size, feeding strategies and other life history traits [4–6]. Large herbivore communities are undergoing rapid changes at global and local scales, including a biased loss of the largest species [1, 7], as well as a loss of trophic interactions and complexity [8–10]. Consequent shifts in the impacts of herbivores on ecosystems are thus expected, but to predict these shifts we first need to understand how the structure of herbivore communities is changing.

Large herbivore assemblages often comprise both wild species and rangeland livestock [11]. Humans have facilitated great increases in the abundance of livestock, which have functionally replaced wild herbivores in many parts of the world [11–13]. Such shifts in herbivore communities are concurrent with other environmental changes such as changing climate patterns [14], woody plant expansions [15] and altered forestry management practices [16]. Such changes can also have differential impacts on livestock and wild herbivore fitness [17]. In light of rapidly changing environments around the world, we need a deeper understanding of how environmental factors, which occur alongside socio-economical factors, drive shifts in herbivore assemblages.

The impacts of large herbivores are known to influence ecosystem processes and interact with biosphere properties [18, 19]. However, herbivore impacts have not been adequately included in models of vegetation-biosphere interactions. The production of spatially-explicit layers detailing herbivore biomass and functional composition (*herbivore biomass surfaces*) can change this [5]. For example, Hempson et al. [12] showed that the replacement of wildlife by livestock in African savannas causes changes in fire frequencies, woody plant cover and greenhouse gas fluxes.

The functional homogenisation of herbivore communities, often caused by the replacement of diverse wild herbivore assemblages with livestock, has partly increased attention on rewilding and the conservation or restoration of trophic dynamics [20]. This is particularly relevant in northern tundra ecosystems where the functional diversity of large herbivores is far lower than during the Pleistocene with subsequent difference in ecosystem regulation [21, 22]. It has been suggested that rewilding of large herbivores could play a role in mitigating climate change impacts in many regions. These include northern European landscapes [23], where opportunities are provided by farmland abandonment [24], and lower competition between wildlife and livestock management [25].

Herbivore populations are affected by environmental dynamics, as well as agricultural policies and hunting quotas, which are set across international, national and regional levels [26]. Over the second half of the 20^th^ century many wild cervid species have expanded in distribution [27, 28]. In Norway, increasing densities of wild browsing cervids have partly replaced grazing livestock in rangelands throughout the country [29]. In combination with steep environmental gradients caused by topography and oceanicity, this makes Norway an interesting case study of the dynamics of co-occurring wildlife and livestock. The ultimate causes of these changes are often socio-economic. For example, intensified agricultural production has driven lower densities of livestock in rangelands (as livestock are predominantly housed in pastures and indoors [30]), while changed forest management practices and demographically-targeted hunting policies both affect populations dynamics of wildlife (e.g.[31, 32]). Socio-economic drivers of change in large-herbivore communities are hierarchical, with agricultural and hunting levels influenced by international, national and local (i.e. county or municipality) authorities [30, 31]. However, the impact of these ultimate drivers are likely to be modified by environmental variables through the uneven distribution of natural resources and differences in cultural histories [33]. It is therefore uncertain, how environmental variables will affect large herbivore communities in space and time.

In order to integrate management of wild herbivores and livestock, it is necessary to identify appropriate management regions and scales, as well as areas where there has been rapid changes in herbivory regimes. In this study, we aim to map large herbivore biomass surfaces in Norway across a 66-year period up until 2015, and characterise herbivore assemblages in space and time. We explore which regions of Norway have seen greatest shifts in herbivore community composition and test the hypothesis that the change in wildlife biomass is negatively related to the change in livestock biomass due to competition [25]. This information will be critical in developing a full understanding of both the ecological and social consequences of changes in herbivore communities, and developing solutions to future management of wild and domesticated herbivores.

## Methods

The study system used here is unenclosed land within Norway (*utmark* in Norwegian). Species of large herbivores (here defined as species >10 kg) using unenclosed land comprise both wild species and rangeland livestock. The wild species are the three forest cervids: moose (European elk, *Alces alces*), roe deer (*Capreolus capreolus*) and red deer (*Cervus elaphus*) and a population of musk oxen (*Ovibos moschatus*), reintroduced in the 1940s. Reindeer (*Rangifer tarandus*) include both wild and semi-domestic populations that were assessed separately. Four species of livestock use unenclosed land in Norway: domestic sheep (*Ovis aries*), cattle (*Bos taurus*), goat (*Capra aegagrus hircus*) and horse (*Equus ferus caballus*). The wild boar (*Sus scrofa*) has recently re-established in south-eastern Norway. Since its current distribution is highly limited, and abundance low [34], it is not included here. The herbivore species of Norway vary from species which are predominantly grazers (livestock) to browsers (wild ungulates), with red deer, and goat being intermediate feeders in this classification [35]. The herbivores also vary in their use of outlying land [36], with some being associated mostly with forests (moose, roe deer, red deer) and others with mountains (sheep, reindeer, musk ox). Goats, cattle and horses have traditionally used all but the most alpine areas.

Metabolic biomass of large ungulates was calculated for every Norwegian municipality (2017 list, summing data for municipalities that merged during the study period; in the rare case of splitting of a municipality and merging with two others, the biomass followed the larger area) for each 10th year from 1949 to 2009 and in addition 2015. Data were collated from agricultural statistics for livestock, reindeer herding data for semi-domestic reindeer and hunting statistics for wild cervids. These data sources provide numbers of male and female adults and juveniles and in the case of livestock the numbers that are using unenclosed land (for full details see [29]). Sex and age-specific (calves, adults) numbers of wild cervids were estimated using a simple population model, which, besides annual harvest, also includes estimates of recruitment rate, mortality rate and population growth rate [29]. Demographic rates were estimated based on hunter observations (recruitment rates), capture-mark-data (mortality rates) and hunting statistics (population growth rate), or extracted from the literature [29]. Animal numbers were converted to metabolic biomass using allometric scaling and standardised for the amount of time spent in unenclosed land (100% for wild herbivores, less for livestock which range during summer only, see [29]). Metabolic biomass of livestock is thus given as an average across the year. Finally, we standardised the metabolic biomass within municipalities by dividing the estimate by the area of unenclosed land (all land, except enclosed land, built-over area, lakes and glaciers). The study system used here had some minor differences from that of Austrheim et al. [29], as cows and heifers are pooled as cattle in the current study, semi-domestic reindeer are classified as livestock, and in addition we include the musk ox.

### Data analysis

For each municipality we calculated the total metabolic biomass of all wildlife species (including wild reindeer) and livestock species (including semi-domestic reindeer) separately, and subsequently found the proportion of total biomass comprised by wild herbivores (moose, roe deer, red deer, wild reindeer and musk ox). The change in wildlife biomass (biomass difference between 1949 and 2015 per municipality) was modelled as a function of the change in livestock biomass (biomass difference between 1949 and 2015). We also accounted for other factors that may affect change in wildlife biomass, notably climate (temperature and precipitation), land-cover and, due to historical absence of forest cervids from northern Norway, latitude.

Mean temperature of the warmest quarter (summer temperature) and mean annual precipitation were downloaded as *bioclimate* variables from WorldClim [37]. Land cover was summarised as the proportion of total municipality area classified as (i) *agricultural*, (ii) *forest*, and (iii) open-natural vegetated (defined as 'open firm ground'; ground which is not farmland, forest, developed or used for communications purposes, and is for the most part tundra vegetation, and is herein referred to as *tundra*) from the Norwegian Land Cover map *AR50* [38]. Other land-cover types (e.g. mires, urban, freshwater etc) were not used, and the three selected land-cover types were effectively independent of one another. Historic land-cover data was not available, and recent (2016) land-cover was deemed adequate to infer coarse spatial patterns in land-cover across Norway. Pairwise correlations between explanatory variables (**Fig A in S1 File**) showed that mean summer temperature was strongly correlated with tundra vegetation (r = -0.89) and latitude (r = -0.68). For these two variables, we used the residuals of a linear regression of natural vegetation cover against mean summer temperature (or latitude against mean summer temperature) in the models to analyse the importance of these variables after accounting for temperature. This approach assigns priority to temperature over tundra vegetation or latitude. Other independent variables were not strongly correlated (|r| ≤ 0.58, **Fig A in S1 File** and variance inflation factors were all <2.04).

Generalised least square (GLS) models were used to evaluate the relative effects of livestock biomass change on the change in wildlife biomass, in relation to climatic and land-cover variables. All variables were standardised before including them in the models to make coefficient estimates directly comparable. To account for spatial autocorrelation, we fitted exponential spatial covariance structures within the GLS models, using the coordinates of the centroid of each municipality. Since no single model was strongly supported, we used a model averaging approach based on AIC (Akaike Information Criterion) to assess the relative importance of each variable, defined as the summed AICc weights across all models including that variable. Estimated coefficients of each variable were averaged across all models (ranging from a null model to one including all explanatory variables), and weighted according to the probability associated with each model. Models were developed using the R packages *nlme* [39] and *MuMIn* [40].

Herbivore assemblage composition was assessed by hierarchical clustering. We first developed a Bray-Curtis dissimilarity matrix based on metabolic biomass across the herbivore species within each municipality and year. Hierarchical clustering was undertaken using the Ward method, since this approach minimises the total within-cluster variance, such that assemblages within the same cluster are similar in terms of herbivore composition. The resulting cluster dendrogram (**Fig B in S1 File**) was cut to give the optimum number of clusters (Krzanowski and Lai index, within the NbClust package [41]). No wild herbivores or livestock were recorded in two municipalities during 2009 and 2015, these were omitted from the cluster analyses for all years. The species composition of each cluster was visualised, and the geographic distribution mapped over time to understand spatial-temporal change in herbivore assemblages, and along temperature and precipitation axes to visualise the shifts in assemblage in environmental space. Finally, we used principle response curves [42] to assess the temporal dynamics of the total Norwegian herbivore community against a baseline of the first year (1949), and non-metric multi-dimensional scaling analysis to visualise herbivore assemblages over the combination of time and space. The latter two approaches were executed in the *vegan* R package [43]

## Results

**Fig 1:**
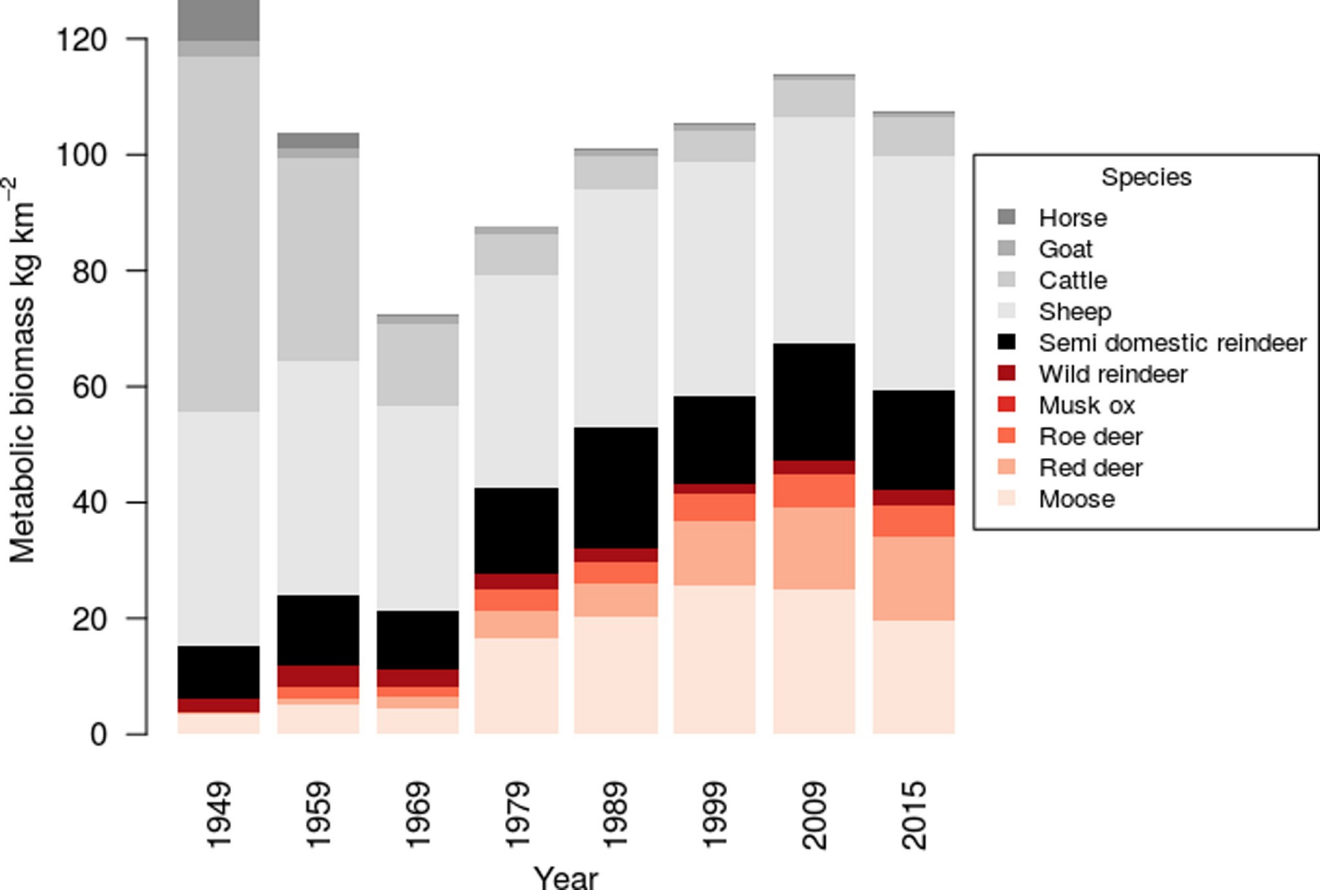
Metabolic biomass (kg km^-2^) of large herbivores across Norway between 1949 and 2015. Livestock species are shown in shades of grey and wild herbivore species in shades of red.

Total large herbivore metabolic biomass across Norway decreased from 127 kg km^-2^ (unenclosed land) in 1949 to a minimum of 72 kg km^-2^ in 1969. After this it increased again to 113 kg km^-2^ in 2009 before declining to 108 kg km^-2^ in 2015 (**Fig 1**). The decrease between 1949 and 1969 was driven by declining livestock metabolic biomass (in particular cattle, **Fig 1**), highest in 1949 at 120 and lowest during 1999 at 62 kg km^-2^. An increase in wild herbivore metabolic biomass (from a minimum of 6 kg km^2^ in 1949 to a peak of 47 kg km^-2^ in 2009, **Fig 1**) was behind the rise in total herbivore biomass from 1969 to 2009, and this was largely due to increases in moose and red deer biomass (**Fig 1**). The rate of change in herbivore community was greatest during 1949 to 1979, with lower rates of change during later periods (**Fig 1 and Fig C in S1 File**).

**Fig 2:**
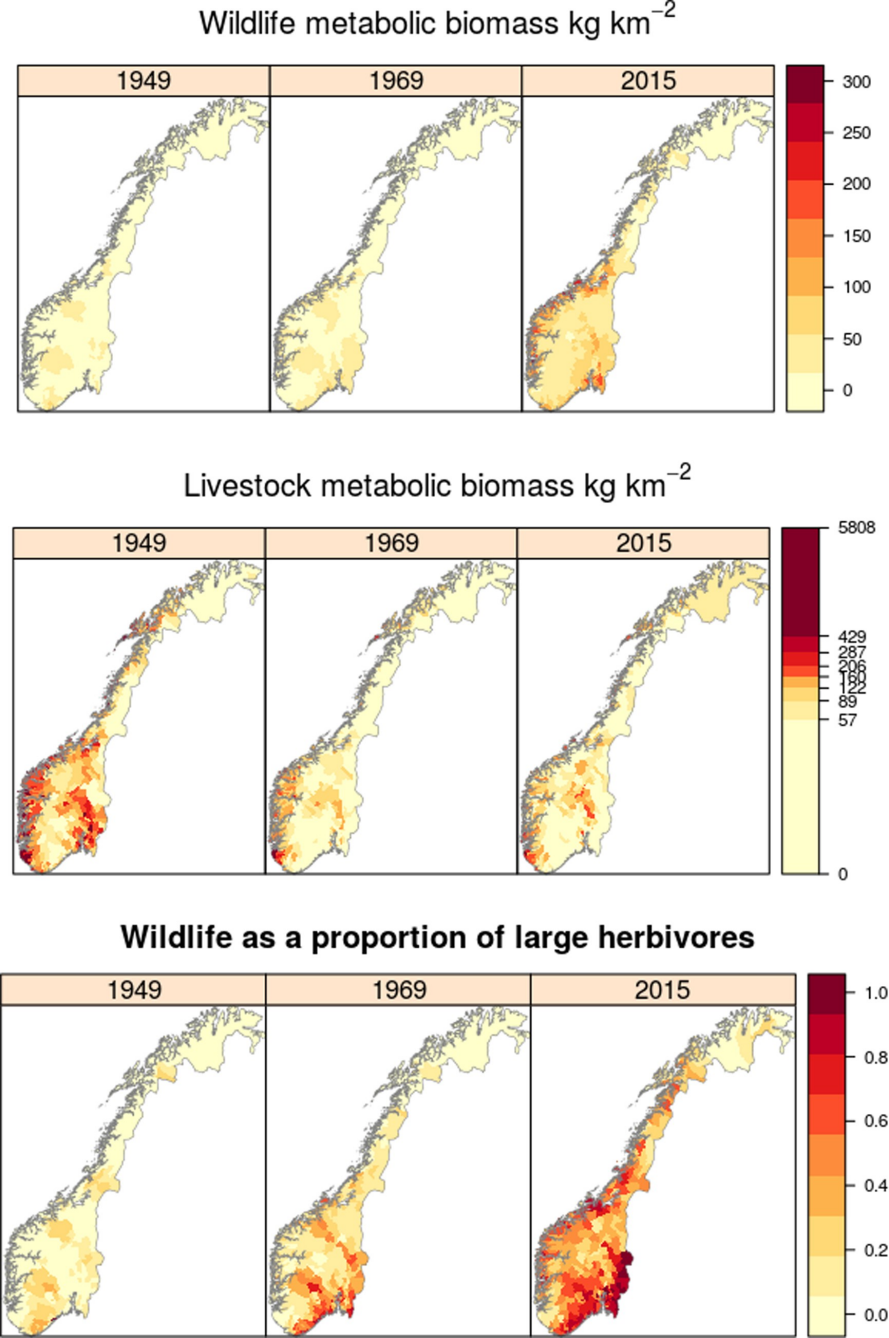
Spatial patterns in wildlife and livestock metabolic biomass across Norway. Metabolic biomass shown for Norwegian municipalities in 1949, 1969 and 2015 (years selected as the maximum and minimum total metabolic biomass, and the most recent year respectively, **Fig 1**). Wildlife metabolic biomass is shown as a proportion of total herbivore metabolic biomass in the bottom row. Note that livestock biomass is shown on a log scale. Data for all years is presented in **Figs E-G in S1 File**.

Spatial variation in total large herbivore metabolic biomass was high and concentrated in coastal and south-eastern Norway (**Fig 2 and Fig D in S1 File**). Wildlife metabolic biomass peaked in south-eastern and central-coastal areas of Norway in later years (**Fig 2A and Fig E in S1 File**). Livestock biomass was highest across the whole of the west region of Norway and lowland eastern areas in 1949, but by 2015 remained high only in the far southwest and inland regions of mid to southern Norway (**Fig 2B and Fig F in S1 File**). In 1949, livestock dominated the large-herbivore assemblages across the whole of the Norwegian unenclosed land and wild herbivores comprised only a median of 1.6% (interquartile range of 0 to 5.4%) of large-herbivore metabolic biomass across municipalities (**Fig 2C and Fig G in S1 File**). However, wildlife became a far larger component of the Norwegian landscape in terms of metabolic biomass since 1969 (>40%, **Fig G in S1 File**), and by 2015 a median of 48% of large herbivore biomass was comprised of wildlife across municipalities (**Fig 2C**, interquartile range 27–67%).

**Fig 3:**
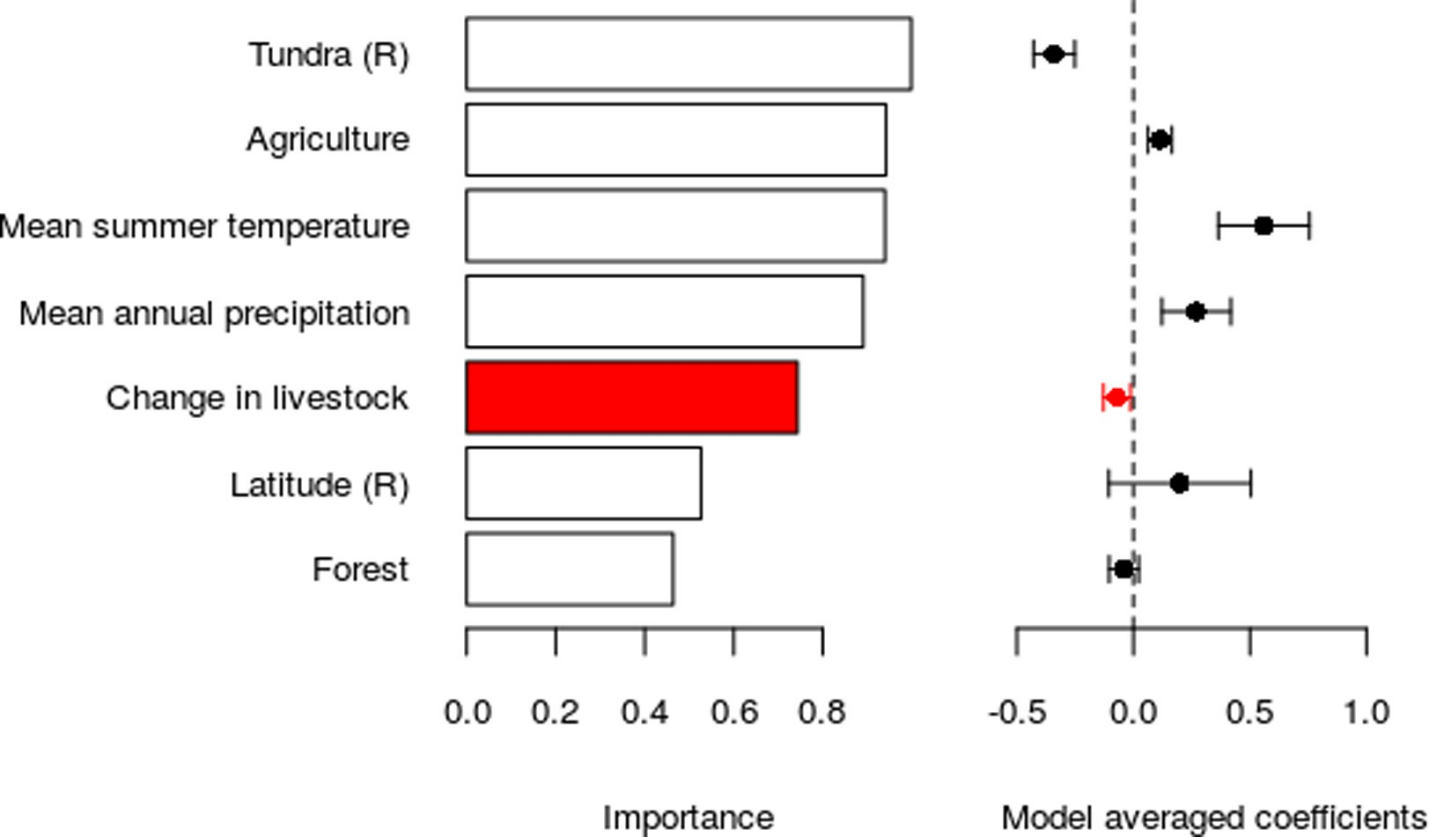
Relative variable importance and model averaged coefficients showing relationships between change in wildlife metabolic biomass and change in livestock metabolic biomass. Output from model averaging on GLS models including an exponential spatial auto-correlation structure. The change in livestock variable is highlighted in red since it is the main variable of interest; the others are included to account for spatial and environmental variability. Tundra land-cover and latitude are modelled as the residuals of the relationship between each of these and mean summer temperature, and denoted with an (R) in the row names.

Although the change in wildlife biomass was negatively related to the change in livestock biomass (**Fig 3**), the support was not strong (relative variable importance of 0.74, full averaged standardised coefficient of 0.07 ± 0.06, P = 0.22). Other factors, notably climate (greater increase in wildlife biomass in warmer and wetter regions) and land-cover (more positive change in wildlife biomass in agricultural regions, less positive change in wildlife biomass in tundra regions), were more strongly related to the change in wildlife biomass (**Fig 3**). Hence, there was a clear biogeographical divide in the proportion of wild biomass in large herbivore communities. In the far north and in mountain regions there was a low proportion of wildlife throughout the study period (median 32%, 17–47% in 2015; **Fig 2C**), compared to coastal and inland lowland regions where the proportion of wildlife biomass increased during the study period, in 2015 reaching 49% (23–65%) in coastal and 66% (17–89%) in inland-lowland regions. In 2015, sheep and semi-domestic reindeer remain the most abundant large herbivore species in the mountain and northern regions. Metabolic biomass surfaces of all large-herbivore species and all years are included as **Figs H-Q in S1 File**.

**Fig 4:**
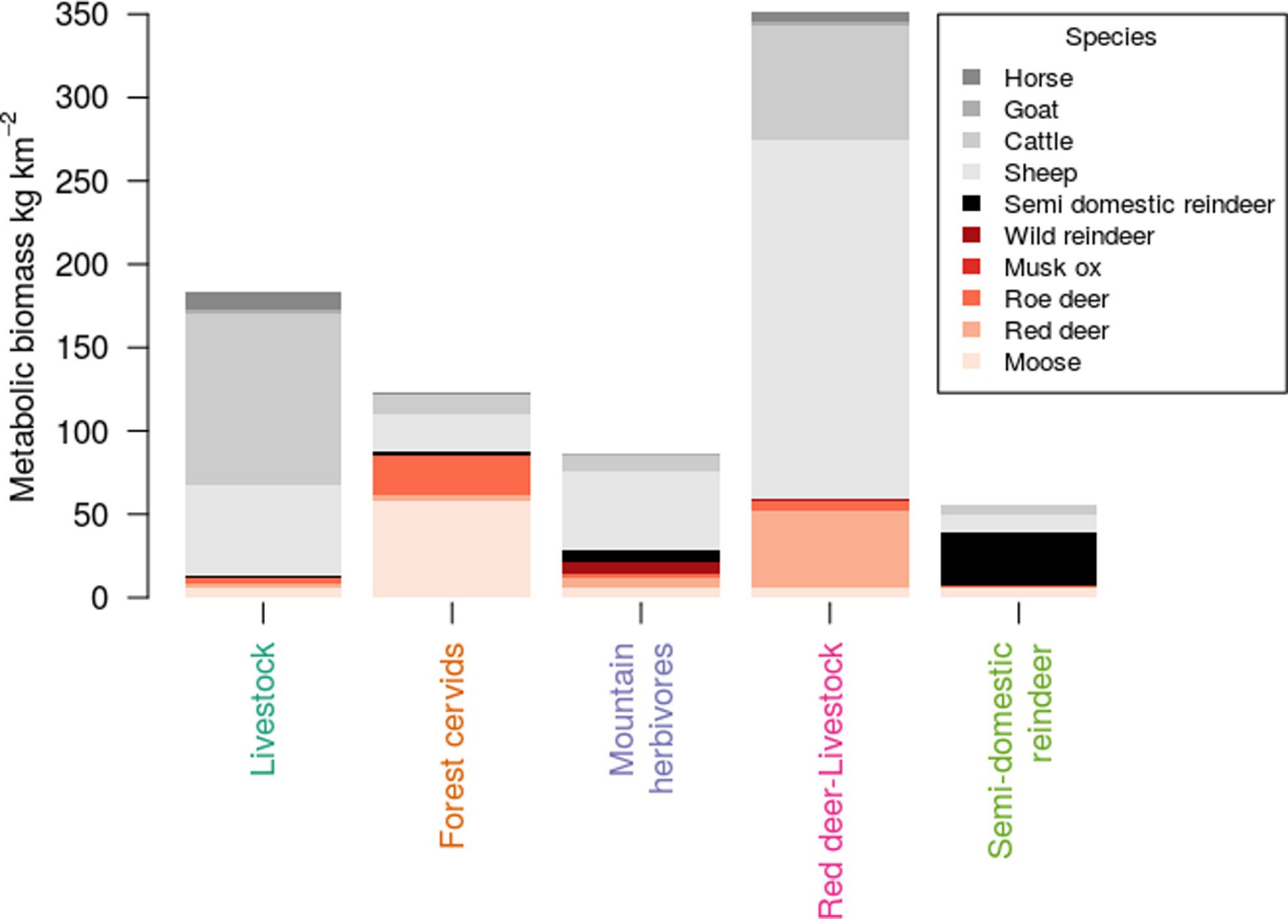
Characterisation of five clusters of herbivore communities across Norway during 1949 to 2015. Assemblages are defined from clustering a Bray-Curtis dissimilarity matrix. Stacked bars show the mean metabolic biomass of each herbivore species in each cluster. Livestock are shown in shades of grey and wild herbivores in shades of red. Community names are coloured to match **Fig 5**.

Norwegian herbivore assemblages over the period 1949 to 2015 grouped into five distinct clusters (**Fig 4 and Fig B and Fig R in S1 File**). One assemblage, termed the **‘Forest cervid’** type, was dominated by obligate browsers, notably moose and roe deer, but also with lower abundances of red deer. The other assemblages were either dominated by livestock (the assemblages named **‘Livestock’** and **‘Semi-domestic reindeer’**) or combined species of both wildlife and livestock (the **‘Mountain herbivore’** assemblage including sheep and both semi-domestic and wild reindeer, species predominantly found in the alpine zone, and the **‘Livestock-Red deer’** assemblage, **Fig 4**).

**Fig 5:**
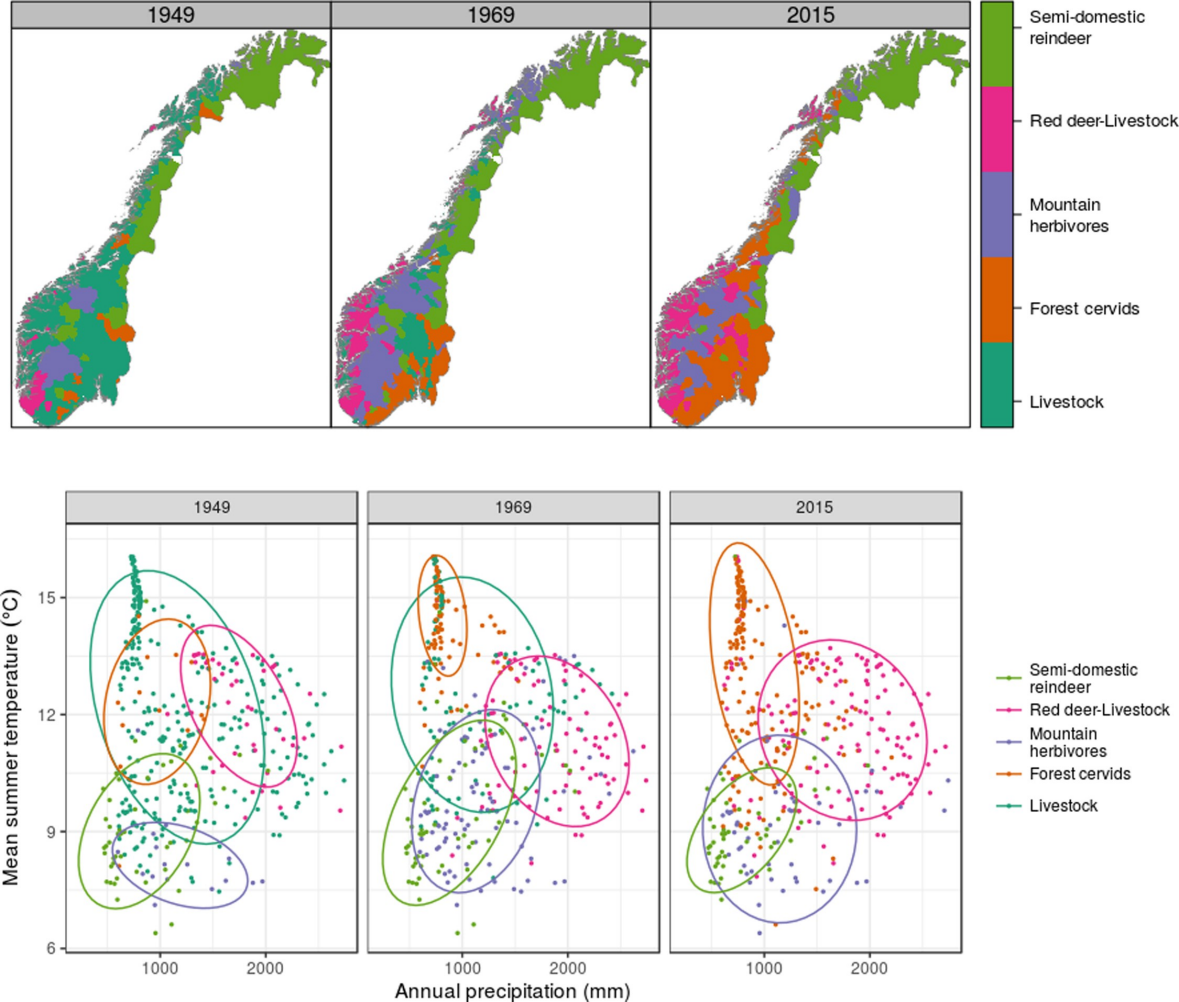
Geographic and climatic distribution of herbivore assemblages across Norway. Top row: the distribution of the five herbivore community clusters presented in Fig 4 across Norwegian municipalities in 1949, 1969 and 2015 (for all years, see **Fig S in S1 File**). White shows municipalities (n = 2) with no herbivore data in 2015. Bottom row: modified Whittaker plots showing the distribution of each municipality in terms of average annual precipitation and summer temperature (note that temperature increases up the y-axis). Points are coloured by the herbivore assemblage characterising each municipality in 1949, 1969 and 2015 (for all years see **Fig T in S1 File**). Ellipses show the 75% quartile of municipalities within each assemblage type.

At the start of the study period, the Livestock and Semi-domestic reindeer assemblages were the most geographically widespread (**Fig 5**). The greatest change in herbivore assemblages between 1949 and 2015 was the decline in the Livestock assemblage in central and southern Norway. In the southeast and central Norway, the Livestock assemblage was replaced by the Forest cervid assemblage (**Fig 5**). The Mountain herbivore assemblage increased in distribution within the mountainous areas of Norway (**Fig 5**) while the Semi-domestic reindeer assemblage showed a slight increase in distribution in central Norway and continued dominance in the north. On the west coast of Norway, the Livestock assemblage was rapidly replaced by the Livestock-Red deer assemblage. The Livestock assemblage spanned the greatest variation of precipitation and temperature during 1949 (Fig 5). This assemblage contracted into warmer and drier regions of Norway by 1969 and completely disappeared by 2015 (**Fig S-T in S1 File**). In wetter regions, the Livestock assemblage was replaced by the Livestock-Red deer assemblage. The Livestock assemblage was replaced by the Mountain herbivore assemblage in colder regions and by the Forest cervid assemblage in warmer and drier regions (**Fig 5**).

## Discussion

Shifts in herbivore community composition have profound impacts on community and ecosystem dynamics. Livestock abundances have increased to dominate herbivore assemblages in most parts of the world [11]. Here we have shown a reversal of this process, where herbivore communities in Norway have reverted from a livestock dominated state around 1949 (associated with widespread use of unenclosed land for grazing during summer months and low cervid densities due to heavy hunting pressure), to a wild herbivore dominated state today. We found that the increase in wildlife biomass was highest in warmer and wetter regions of Norway. Declines in livestock biomass were a modest predictor of wildlife increases, suggesting that direct competition with livestock has not been a major limiting factor of wild herbivore populations over the past decades.

We hypothesised that wildlife dynamics would be driven by livestock biomass due to competition, predicting that changes in wildlife biomass would be negatively related to the change in livestock biomass. This prediction was not supported. There were other more important variables relating to the change in wildlife biomass, notably the cover of tundra vegetation (negative) and agricultural land (positive) and climate, with greater increases in more warmer and wetter regions.

Our study does not find strong support for competition between wild herbivores and livestock as limiting wildlife biomass increase in Norway, as has been suggested for tropical systems [44, 45]. This could be as competition between wildlife and livestock differs at a species level. For example, niche overlap is greater between moose and sheep than moose and cows within Norway [36], so the decrease in cattle (**Fig 1**) did not greatly change the competitive environment for moose. It is more likely that the increases in wild herbivore biomass are due to shifts in hunting management (e.g. changes in total harvest or harvest induced alterations to demographic structure, [46]), or changes in vegetation productivity driven by either climate [15] or non-livestock focussed land management (e.g. forestry [47]).

Although there was no evidence for direct competition between livestock and wildlife driving changes in wildlife biomass, there may be an apparent facilitation (*c*.*f*. apparent competition) of livestock grazing on the population growth of cervids. In Norway, predator populations are managed to be very low, and limited to restricted regions [48]. This management practice is, to a large extent, motivated by the protection of livestock in unenclosed land. In the absence of livestock grazing in outlying land, carnivore densities would likely be far higher in parts of Norway, causing substantially higher predation pressure and limit population growth of cervids. Indeed, the change in composition of livestock in unenclosed land in Norway (i.e. from cattle to sheep, particularly in eastern Norway where predators are most abundant, **Fig 5 and Figs N-O in S1 File**), may even increase the motivation for management to restrict the range and densities of predators, since free-ranging sheep are more vulnerable to predation than cattle [48]. Furthermore, apparent interactions between livestock and wild herbivores can occur through vegetation. Shrubification of alpine landscapes has been shown to have more negative impacts on forage availability of livestock than wildlife, when the livestock dietary niche breadth is narrower [17]. Reduced densities of livestock have been shown to increase tree and shrub plant cover in low alpine regions of Norway [49] and this is also likely to be more beneficial to the browsing cervids than dietary-generalist livestock species.

The finding that northern and mountain regions have not undergone a strong shift from livestock to wildlife dominance demonstrates the continuity of sheep farming in southern mountain regions and reindeer herding in northern areas. This suggests that upland rangeland grazing livestock production systems have been more robust to socio-economic changes than lowland ranging grazing systems [50]. The reversion from livestock to wild herbivores was strong in municipalities with high agricultural-land cover (and it is in these municipalities that livestock decreased most (**Fig A in S1 File**). It is unlikely that land-abandonment in the second half of the 20^th^ century [24] drove this, as we used recent land-cover maps. Instead, this may reflect a shift from an extensive to intensive livestock farming in lowlands, and a shift from animal husbandry to cereal production in South-East Norway. First, in lowland regions livestock, and in particular cattle, are increasingly housed indoors and in fenced pastures [30], in contrast to continued extensive use of uplands for sheep and reindeer grazing [50–52]. Secondly, agricultural areas used for animal fodder (hay and silage) production were converted to cereal production during the 1950s in the climatically favourable regions [53].

A livestock dominated community was the most prevalent herbivore assemblage across Norway in 1949 (**Fig 4**) with broad geographical and environmental range (**Fig 5**). The assemblage that replaced this community over time varied with environmental conditions. In cooler (mountain) areas, this was another assemblage partly dominated by livestock (semi-domestic reindeer and sheep) in addition to wild reindeer, while in wetter areas it was an assemblage including red deer and livestock. In the warmer parts of Norway, an assemblage characterised by forest cervids became dominant. The increase in biomass of red deer and moose within the current range of these species is important, since species distribution models suggest that there will be no increase in suitable range for these species under future climate change scenarios [54]. There is some evidence for livestock having impacts on ecosystem and ecological dynamics that differ from the impacts of wild herbivores (due to different foraging strategies, population densities and seasonality of grazing) [55, 56]. However, the relative impact of different species of herbivores across environmental gradients remains largely unknown and further work is required to understand this.

Spatially-explicit herbivore biomass surfaces have proven valuable in predicting ecosystem dynamics and identifying regions under comparable herbivore regulation due to similar community composition [5, 12]. Such approaches can be expanded upon through integrated mapping of socio-economic aspects of herbivores (e.g. traffic accidents, game meat and forestry) alongside herbivore populations. This facilitates development of holistic management approaches of the system [33]. In our study, we have produced a combined assessment of livestock and wildlife biomass, allowing us to identify spatial and temporal trends in herbivore communities. Although wildlife and livestock are sympatric in many rangelands, they are frequently regulated and managed independently of one another [51, 57]. We suggest that our joint assessment can inspire, and be used as a foundation for integrated management of wildlife and livestock (see [25]) across Norway.

### Rewilding

We have documented a shift from livestock to wildlife metabolic biomass in Norwegian unenclosed land. This can be cast as passive rewilding, defined as a reduction in human control over the landscape [58, 59], although in the Norwegian case, it is a shift in how humans influence the landscape since all wild herbivore populations are under active management, which could be seen as a form of ‘semi-domestication’ [60]. Alternatively the shift in Norwegian herbivore communities could be seen as a form of partial trophic rewilding of herbivore-vegetation interactions [59, 61], since the native species of herbivore increase in abundance and the livestock species decline. However, native large predators are largely absent from Norway, being limited to narrow ranges and low population densities through management [48]. Thus, full cascading trophic dynamics [61, 62] are far from being re-established across Norway.

Trophic rewilding has been proposed as a climate-mitigation measure due to ruminant methane emissions, impacts on vegetation and soils [13, 23]. In Norway, the declining livestock and increasing wild cervids are functionally similar according to the classification of Cromsigt et al. [23]. However, cervids interact with forest management and, given recent population increases, may be viewed as being overabundant [47]. The increase in cervid densities in Norwegian lowlands will alter forest succession following logging [63], reducing ecosystem carbon stocks and affecting albedo [18]. Meanwhile, the maintained sheep and reindeer densities in tundra vegetation will continue to limit treeline advance [49] and shrubification [64, 65] thus limiting aboveground carbon storage [66] but increasing albedo [67]. It has been suggested that trophic rewilding in northern ecosystems could reduce woody plant expansion [21, 23]. However, sheep and semi-domestic reindeer remain numerous in the Norwegian tundra today, fulfilling this role [49, 68] and the dual impacts of large herbivores on carbon storage and albedo need to be understood to predict impacts on the climate system.

Our study provides a multi-decadal analysis of shifts in herbivore composition across broad environmental gradients. However, early rewilding discussions focussed on reintroducing Pleistocene vertebrate assemblages (or surrogotes thereof [69]) following recognition of the importance of mega-herbivores in driving ecosystem state transitions [22]. This demonstrates the need to take a more long-term perspective when considering biotic shifts [70], for example the shift from browsing to grazing herbivores between the Pleistocene and the Holocence [6] and functional paucity of contemporary northern herbivore assemblages [21]. The shifts in Norwegian herbivore assemblage should thus be cast in light of Holocene scale land-use changes (gradual intensification from the Neolithic to the early 20th century, followed by extensification and abandonment [71]), and the colonisation patterns of wild herbivores (a long-term transition from moose to red-deer, driven by both climate and land-use change [72]). The status of the wild boar and musk ox as alien species in Norway, despite their presence in Norway during historic times and the Pleistocene respectively, is also questioned in a rewilding context.

### Conclusions

In this study, we have mapped biomass surfaces of large-herbivores across Norway between 1949 and 2015. Such biomass surfaces are valuable for including herbivores in environmental models (e.g. [12]). Our analyses show a shift from livestock to wildlife dominated assemblages across Norway’s unenclosed land, with the exception of mountain and tundra habitats. The increase in wildlife was not strongly linked to declines in livestock, suggesting that direct competition does not limit wild herbivore populations, and more complex interactions, likely involving socioeconomic changes and predators, are present. Our results show that the herbivore–ecosystem interactions across Norway have become wilder; however, herbivore populations remain regulated by humans, and full trophic-cascading dynamics do not occur. However, since shifts in herbivore composition involve both wild and domestic herbivores, it is clear that livestock and wild herbivore management should be integrative.

## Supporting information

S1 File Supporting figures Fig A-T. (PDF)

S1 Dataset Metabolic biomass of large herbivores by municipality and year. Shape file with data in kg km^-2^.
(ZIP)

S2 Dataset Large herbivore assemblage for each municipality and year. Shape file with key.
(ZIP)
