# Supplementary material for: Long-term changes in northern large-herbivore communities reveal differential rewilding rates in space and time
Source: PLoS One. 2019 May 21;14(5):e0217166. doi: 10.1371/journal.pone.0217166 (PMC6528981; doi:10.1371/journal.pone.0217166)
Supplement: S1 File — (PDF) [file pone.0217166.s001.pdf]

Supporting Information. File S1 Figs A-

Speed et al. Long-term changes in northern large-herbivore communities reveal differential rewilding rates in space and time

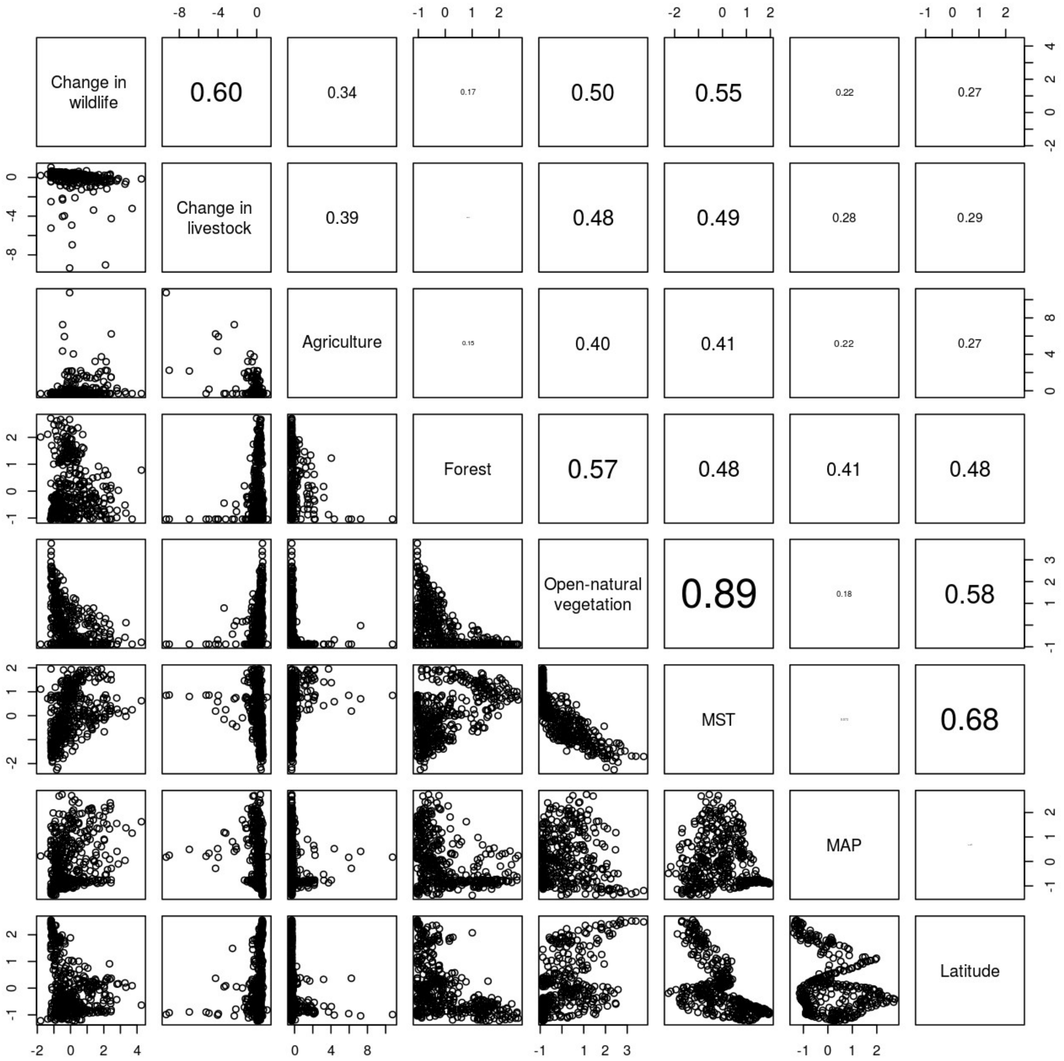

Fig A. Pairwise correlation plots between all dependent and independent variables. The lower panels show pair plots between standardised variables and the upper panels the absolute Spearman correlation coefficient, with text size proportional to the coefficient value.

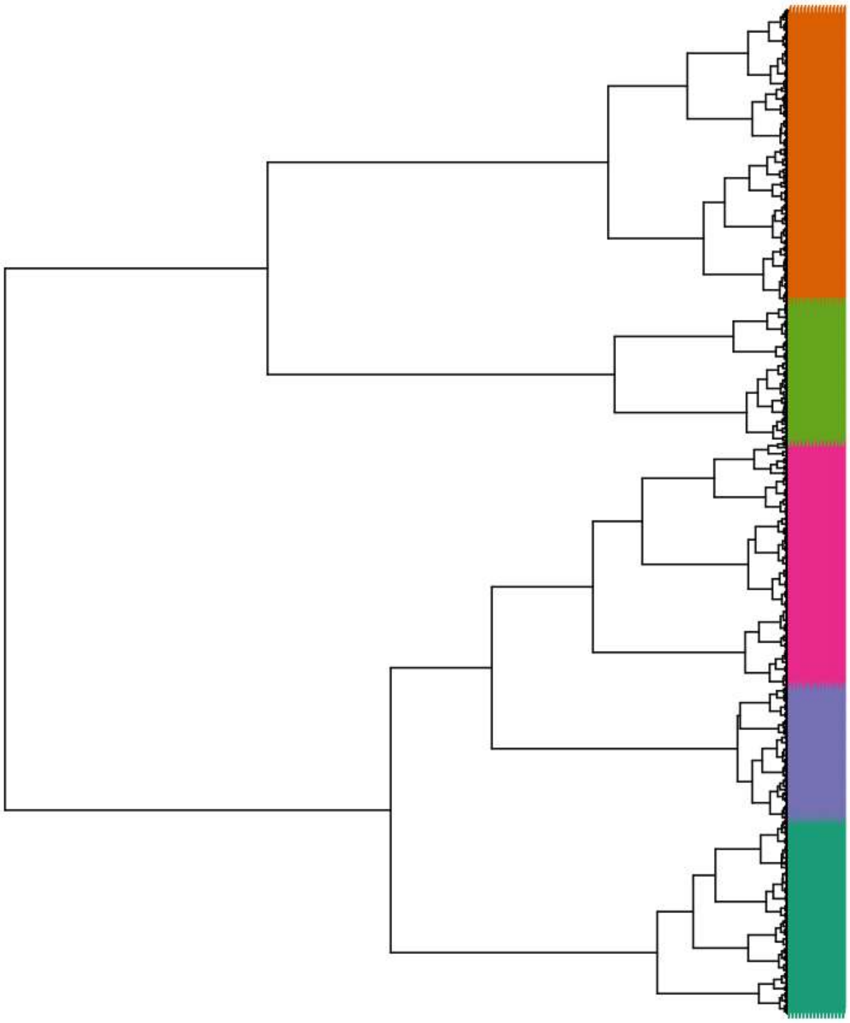

Fig B. Cluster dendrogram of a Bray-Curtis dissimilarity matrix on the herbivore assemblage of each municipality in each year. The dendrogram was cut into 5 clusters (optimal number based on Krzanowski & Lai index), and tip labels coloured.

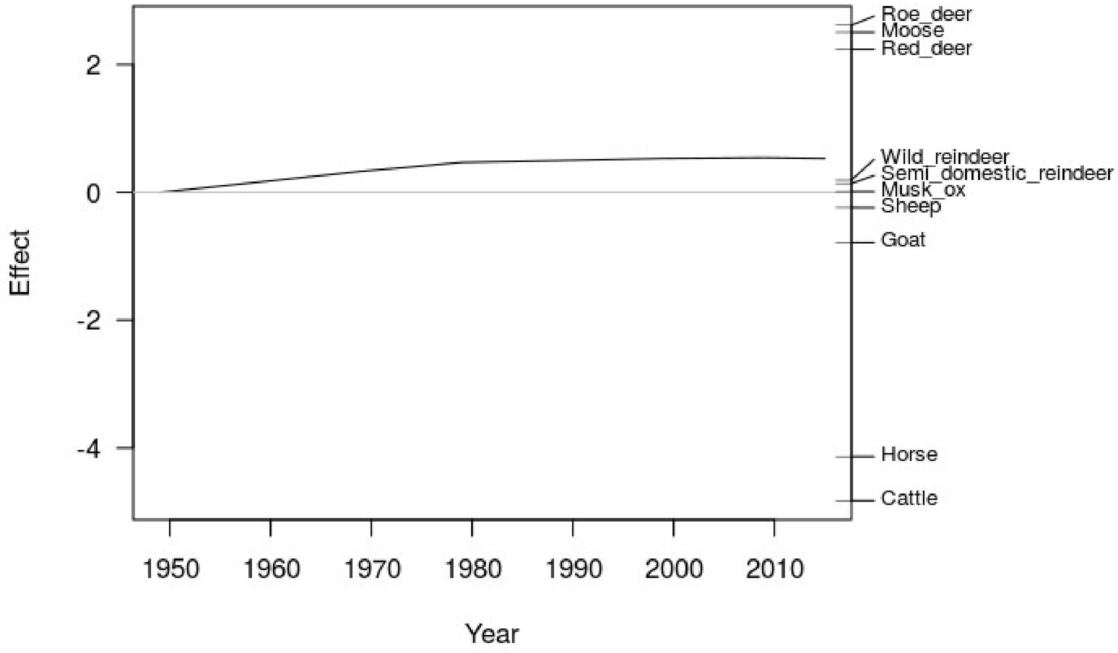

Fig C. Principle response curve showing the shift in the Norwegian large herbivore community along the first axis of an RDA. Positive scores indicate increased relative abundance of wild herbivores. The grey horizontal line at y = 0 represents the 1949 community composition, and the black line the composition of the community at each time point.

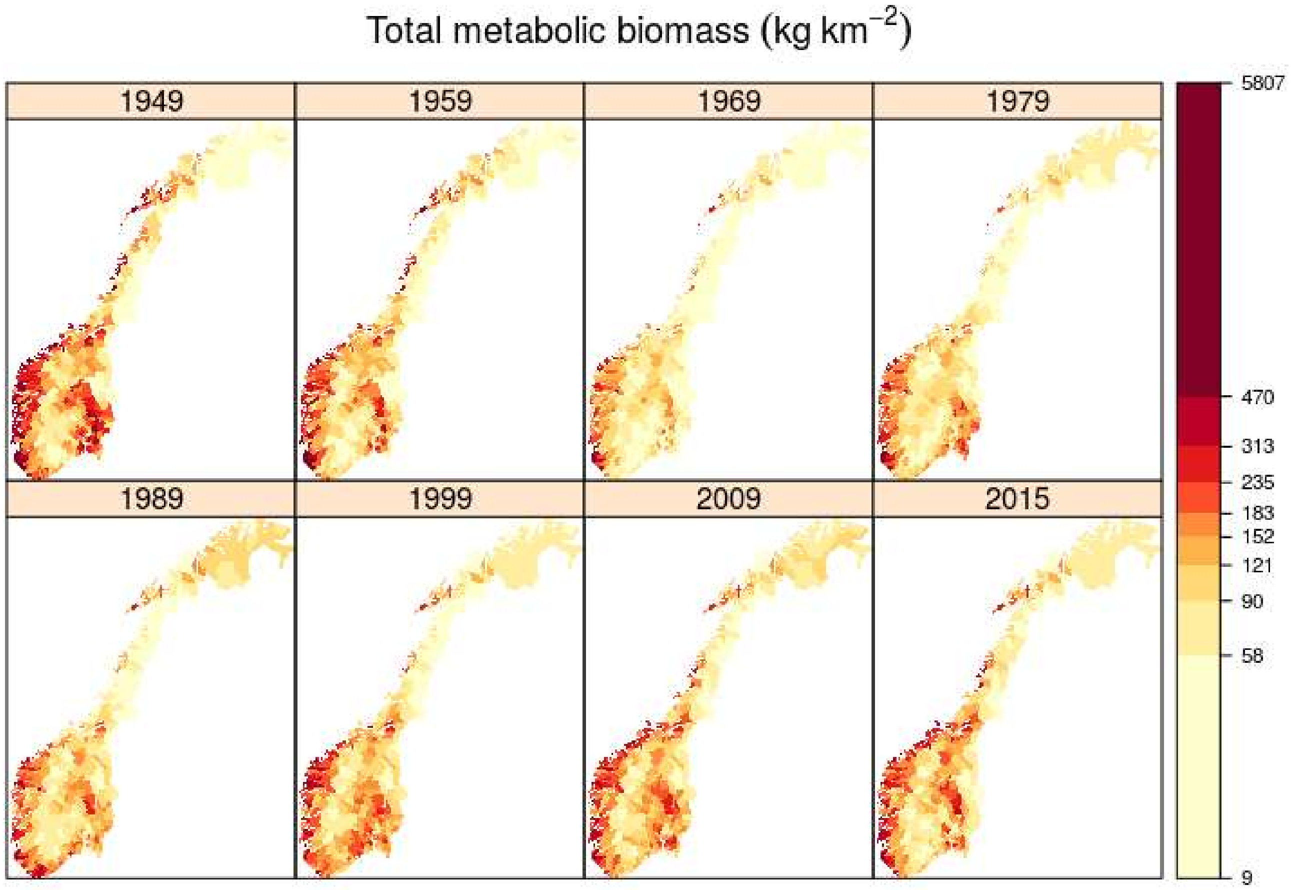

Fig D. Total metabolic biomass (kg km^{-2}) across all years and municipalities in Norway.

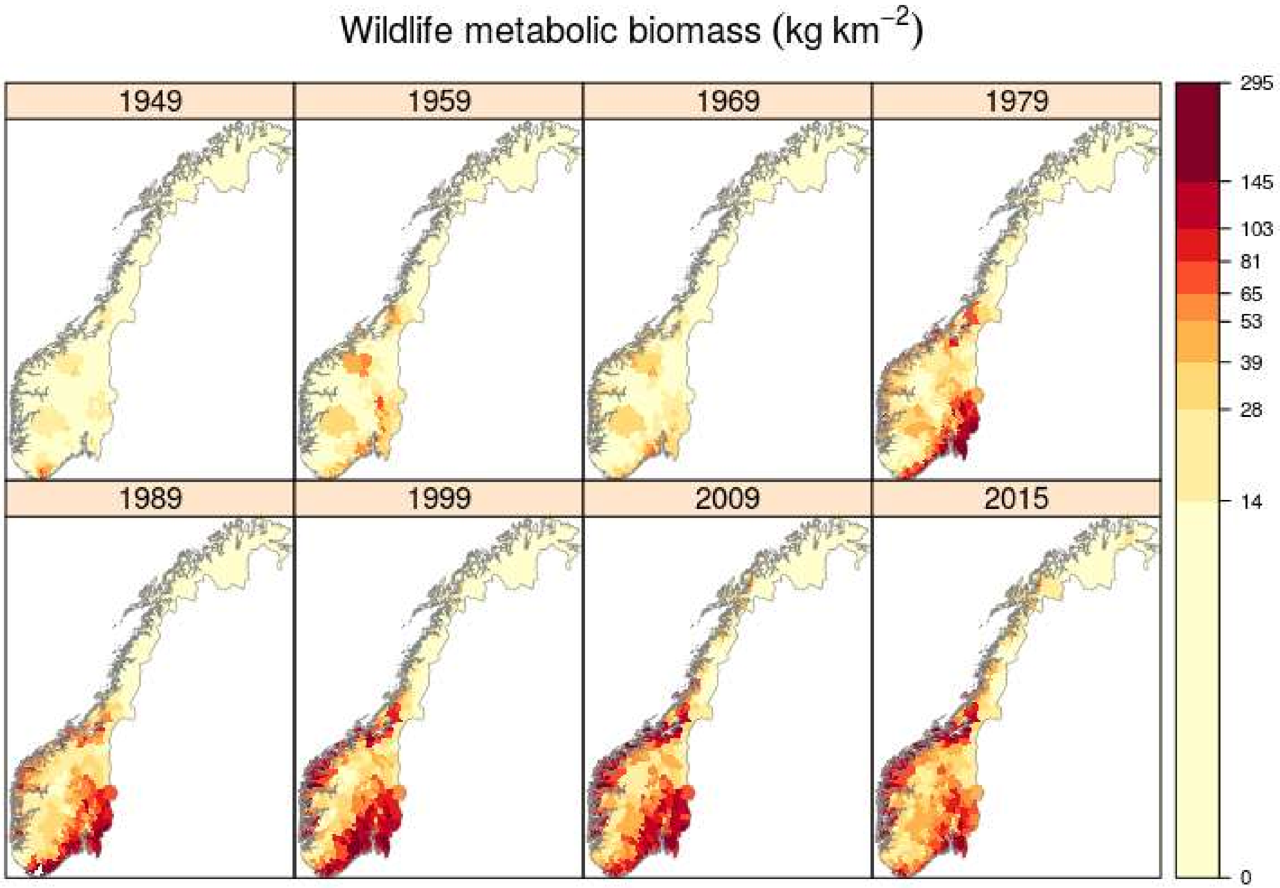

Fig E. Total wildlife metabolic biomass (kg km^{-2}) across all years and municipalities in Norway.

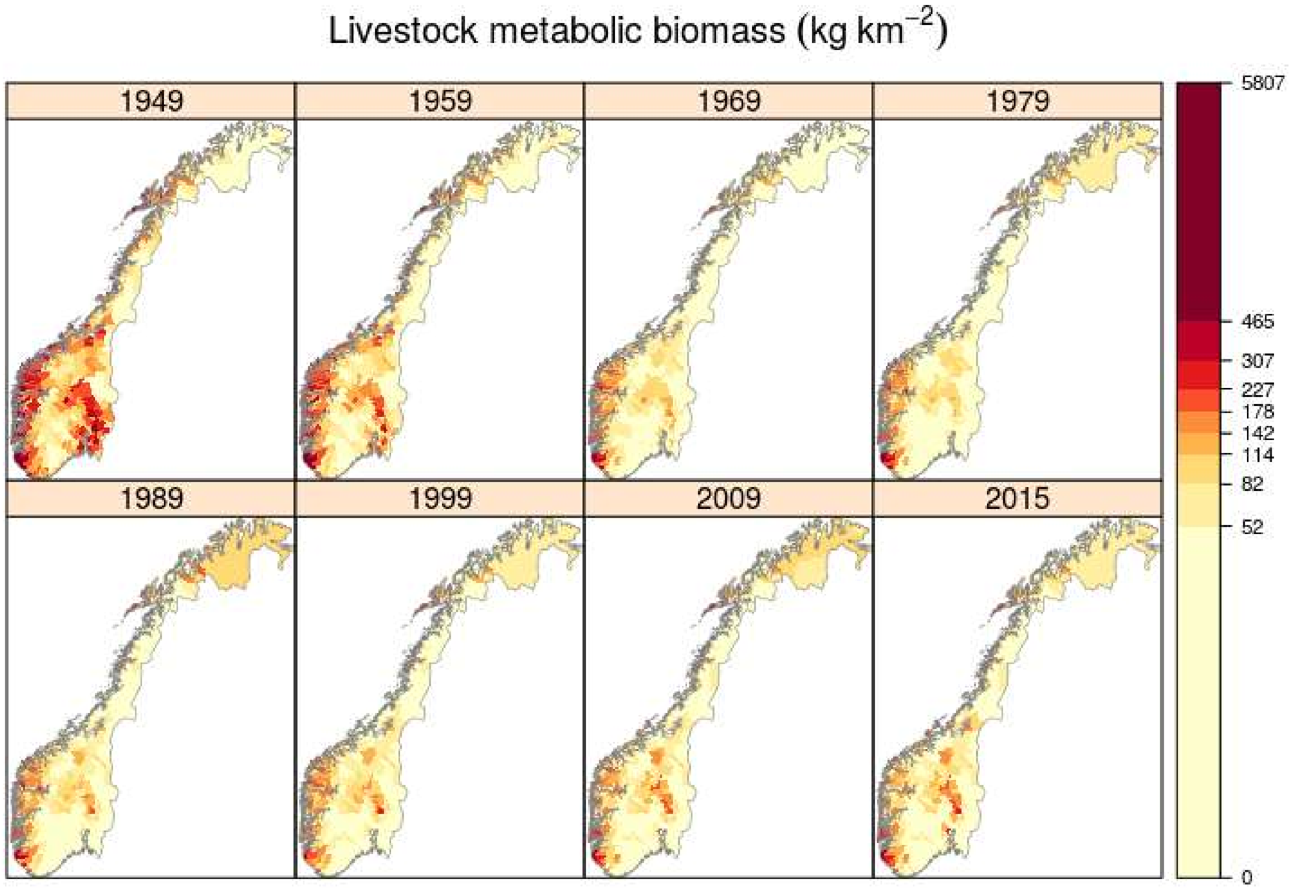

Fig F. Total livestock metabolic biomass (kg km^{-2}) across all years and municipalities in Norway.

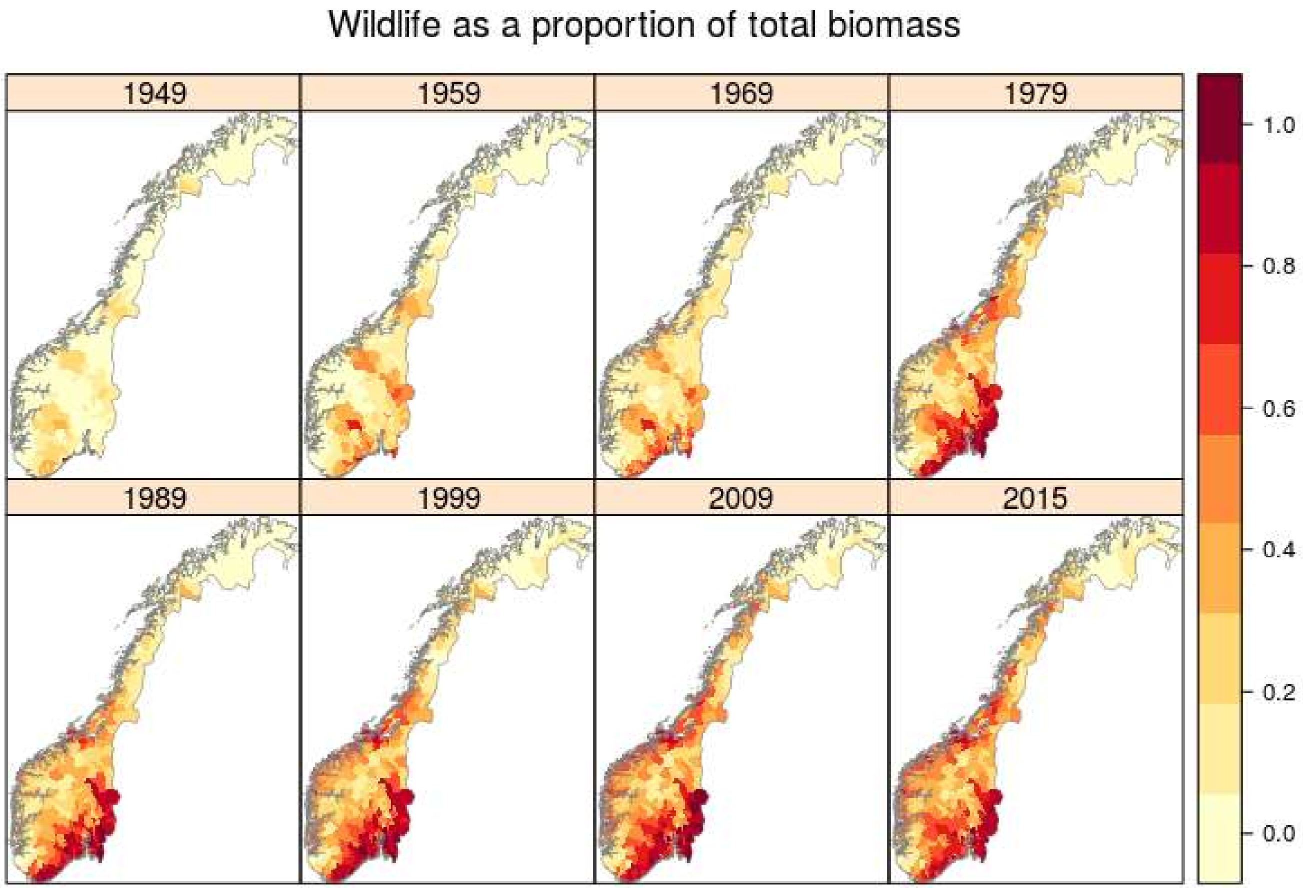

Fig G. Wildlife metabolic biomass as a proportion of total large herbivore metabolic biomass across all years and municipalities in Norway

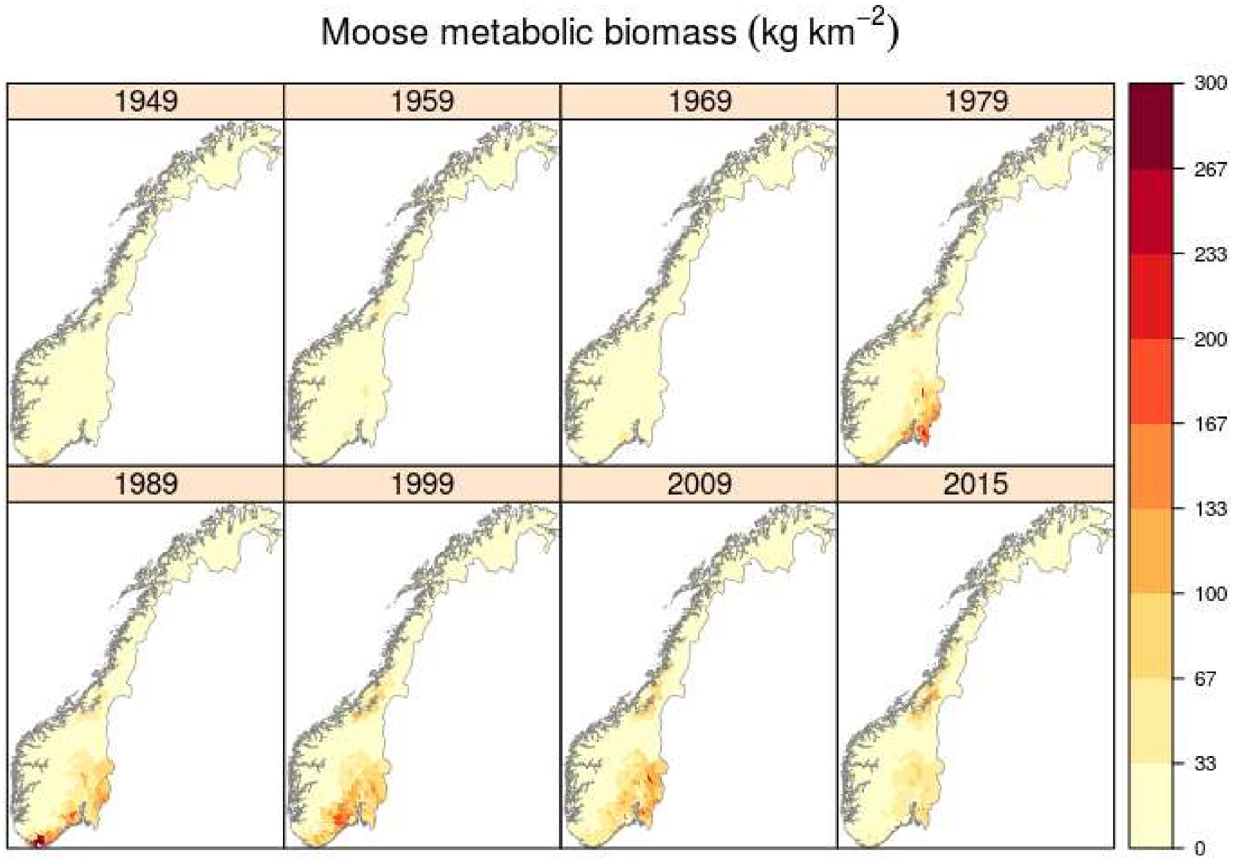

Fig H. Moose metabolic biomass (kg km^{-2}) across all years and municipalities in Norway.

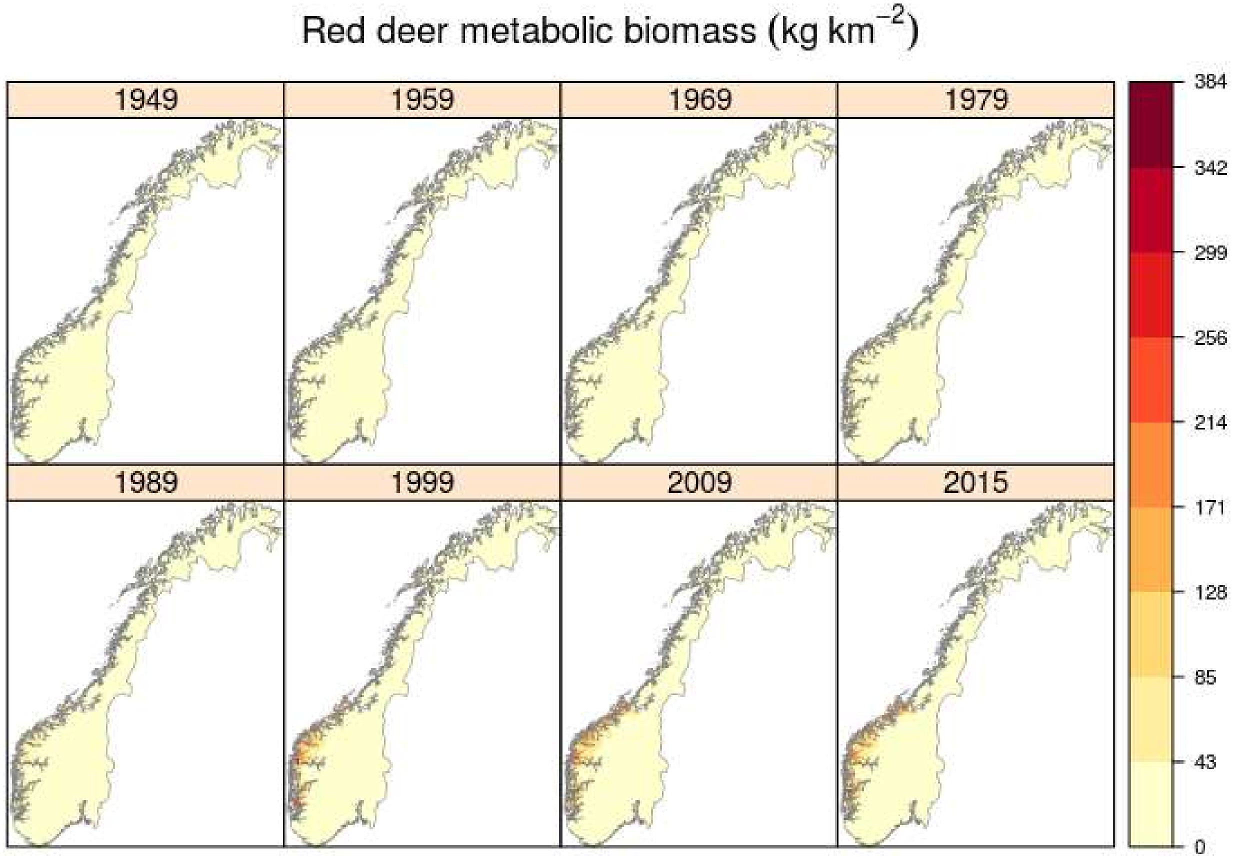

Fig I. Red deer metabolic biomass (kg km^{-2}) across all years and municipalities in Norway.

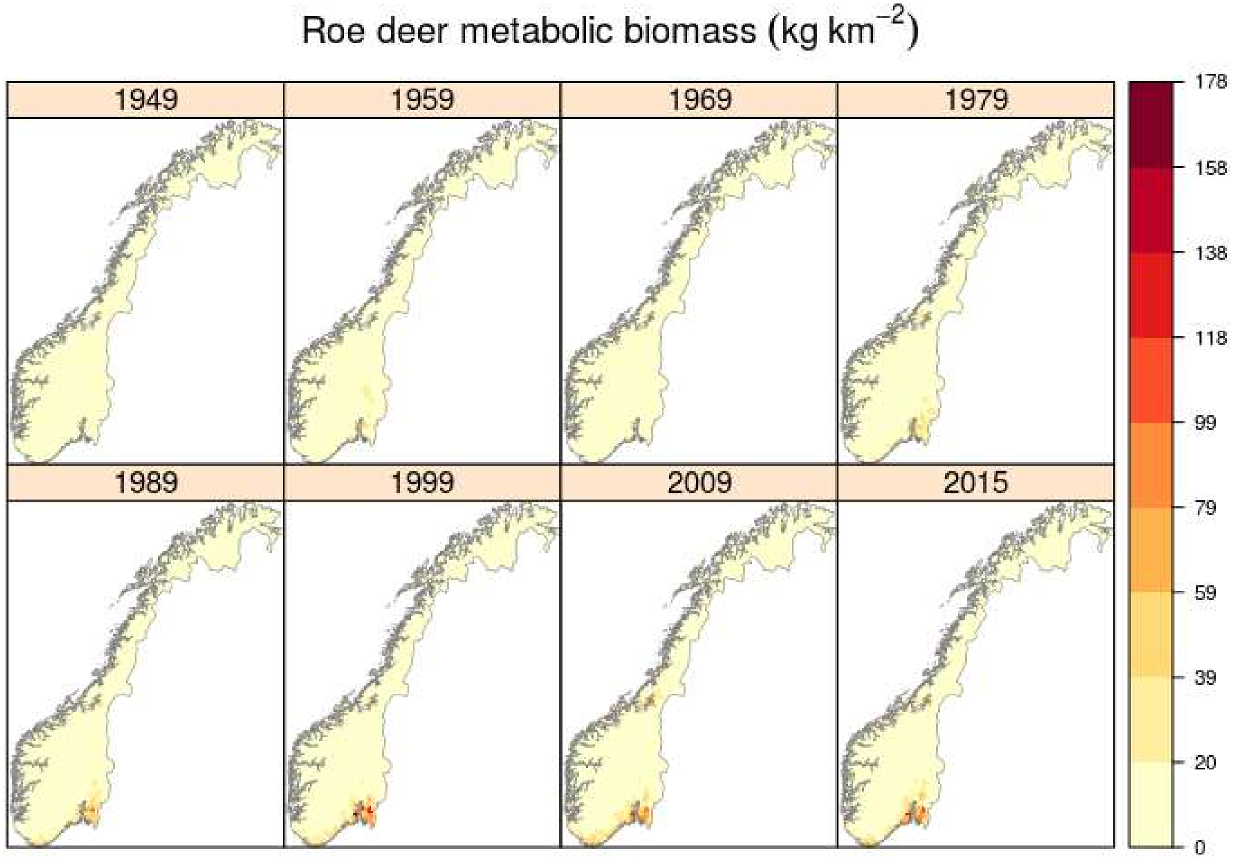

Fig J. Roe deer metabolic biomass (kg km^{-2}) across all years and municipalities in Norway.

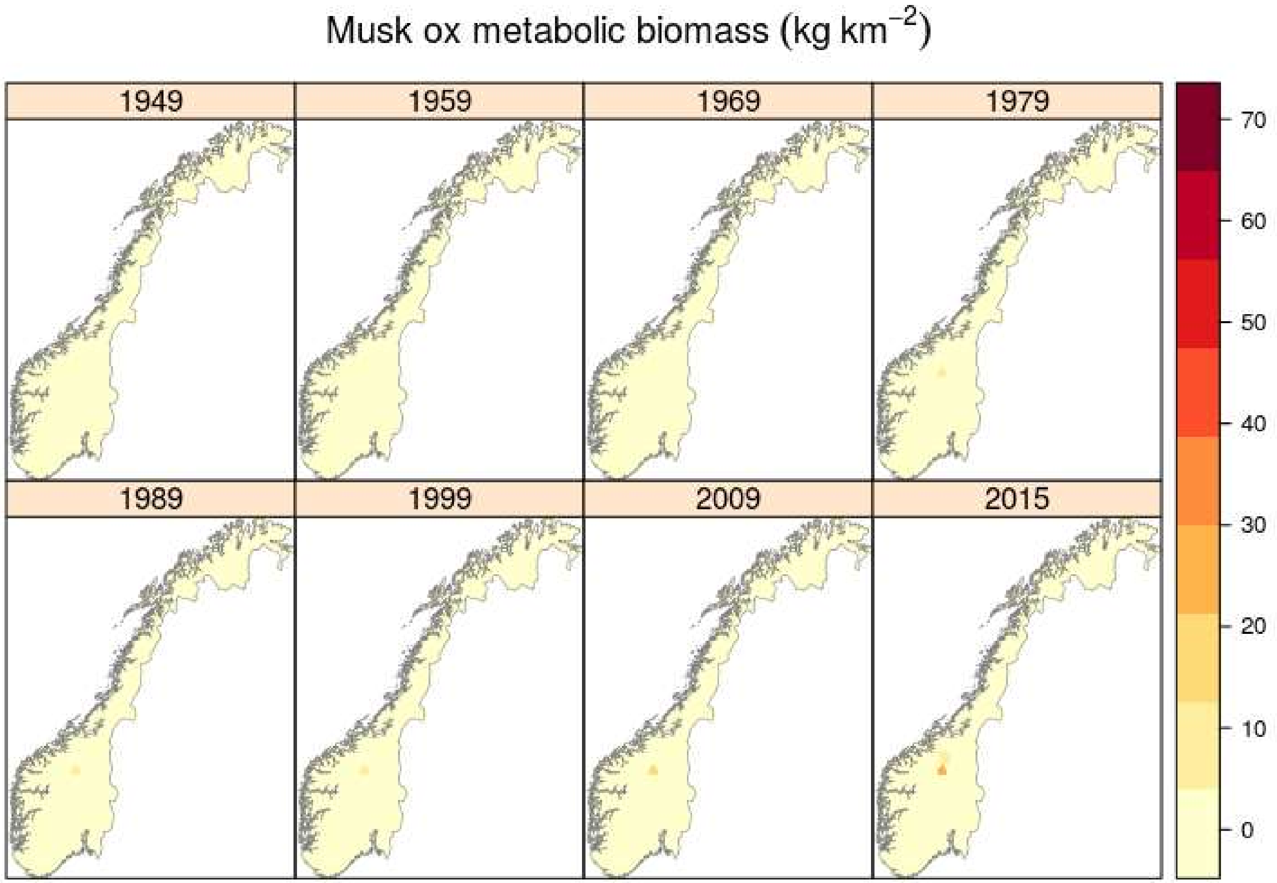

Fig K. Musk ox metabolic biomass (kg km^{-2}) across all years and municipalities in Norway. Note, there are only two municipalities with musk ox present.

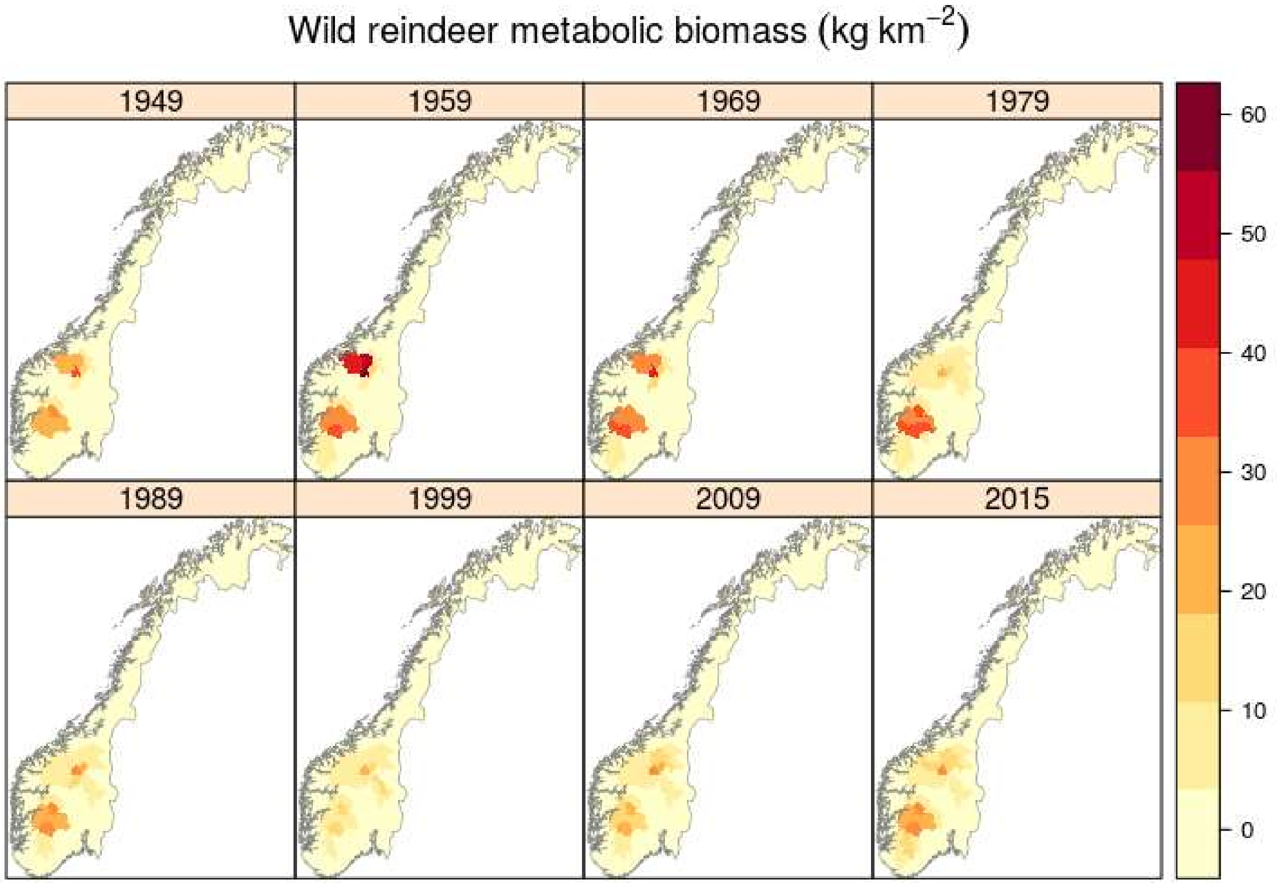

Fig L. Wild reindeer metabolic biomass (kg km^{-2}) across all years and municipalities in Norway.

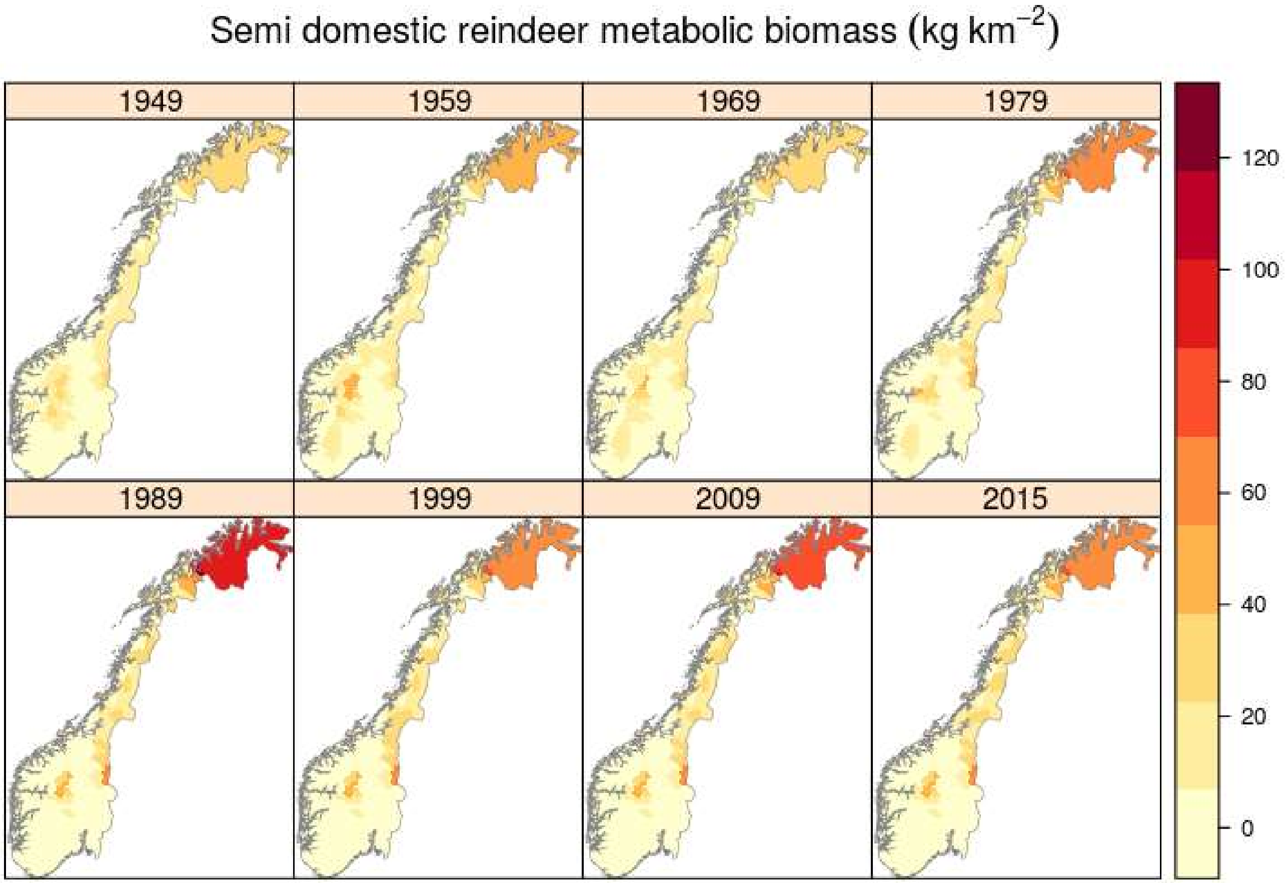

Fig M. Semi-domestic reindeer metabolic biomass (kg km^{-2}) across all years and municipalities in Norway.

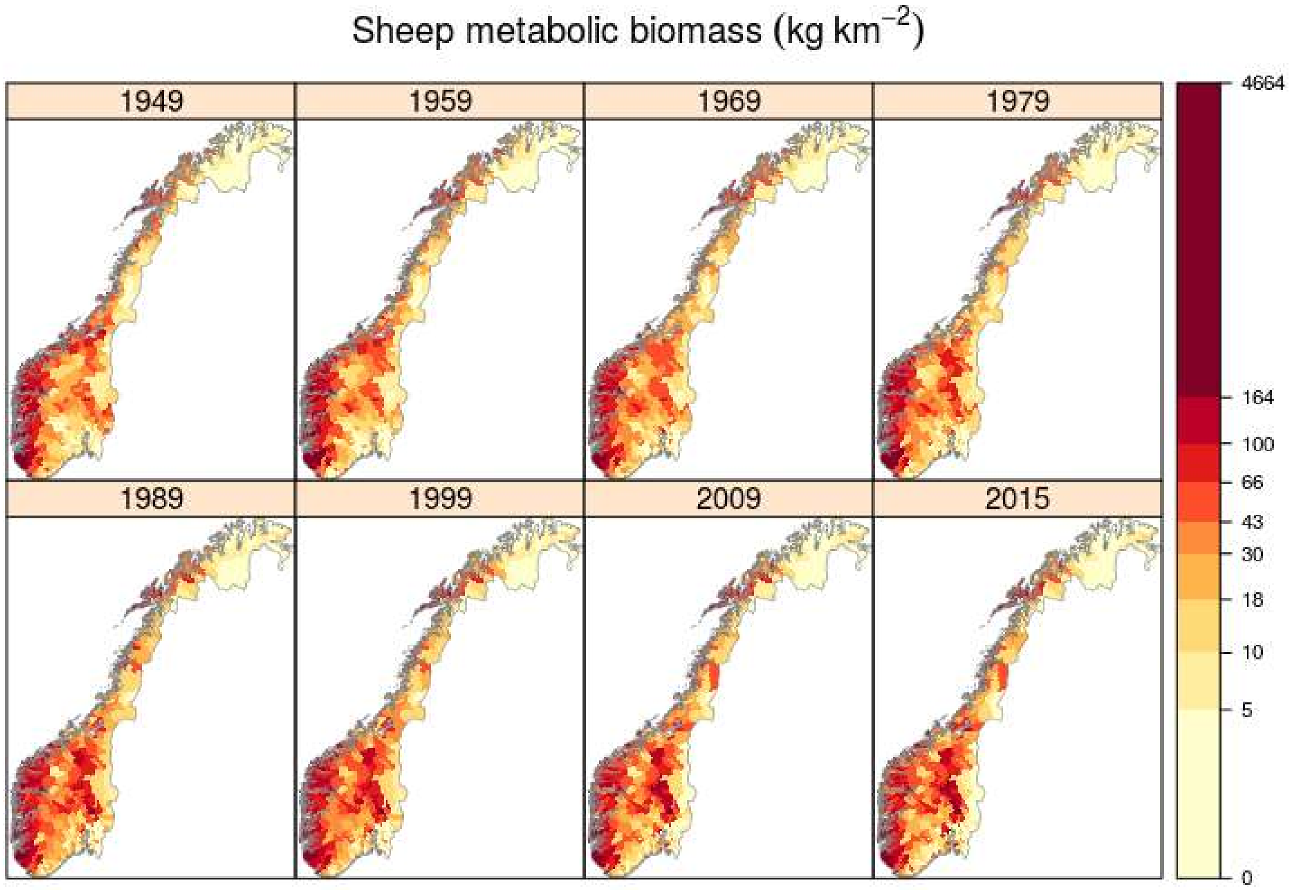

Fig N. Sheep metabolic biomass (kg km^{-2}) across all years and municipalities in Norway.

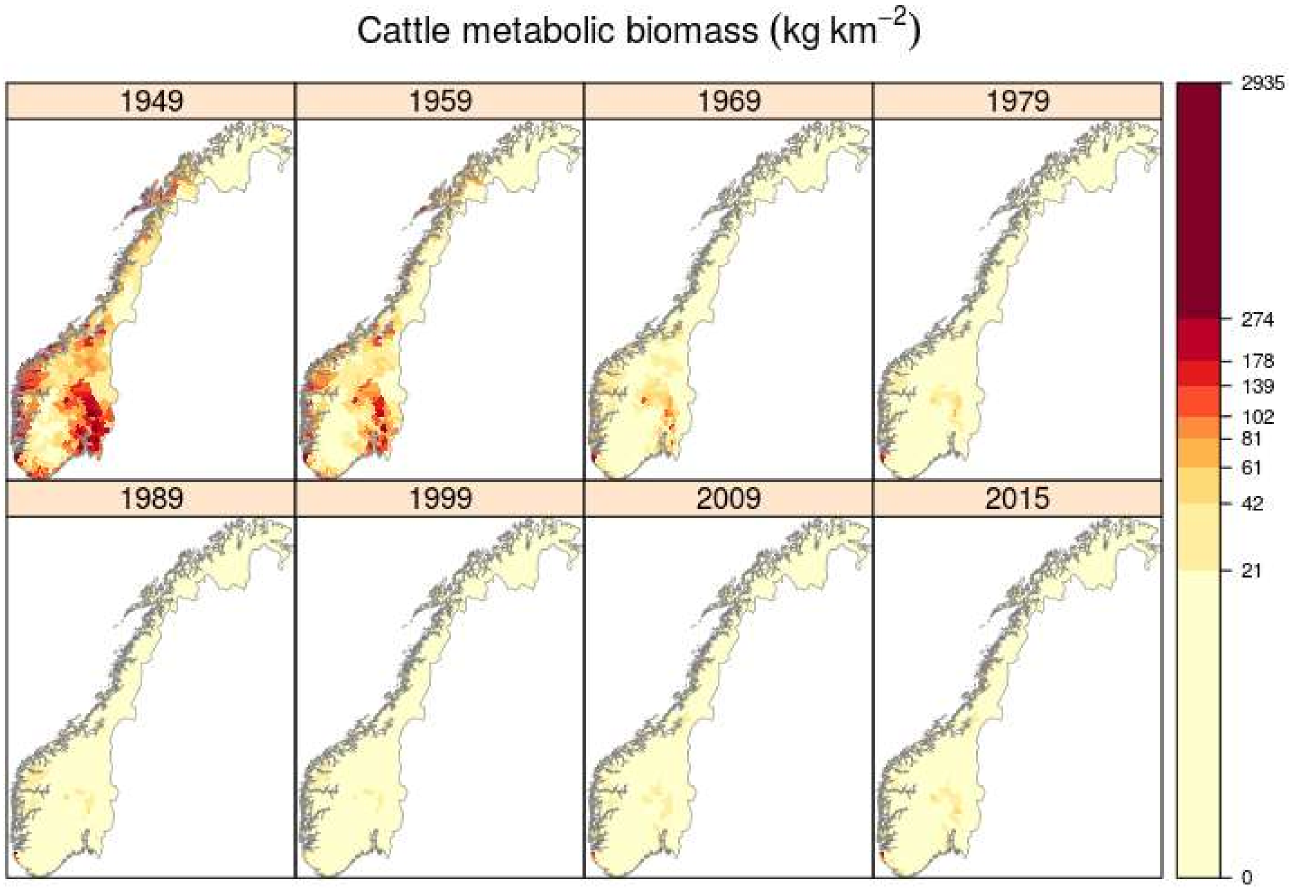

Fig O. Cattle metabolic biomass (kg km^{-2}) across all years and municipalities in Norway.

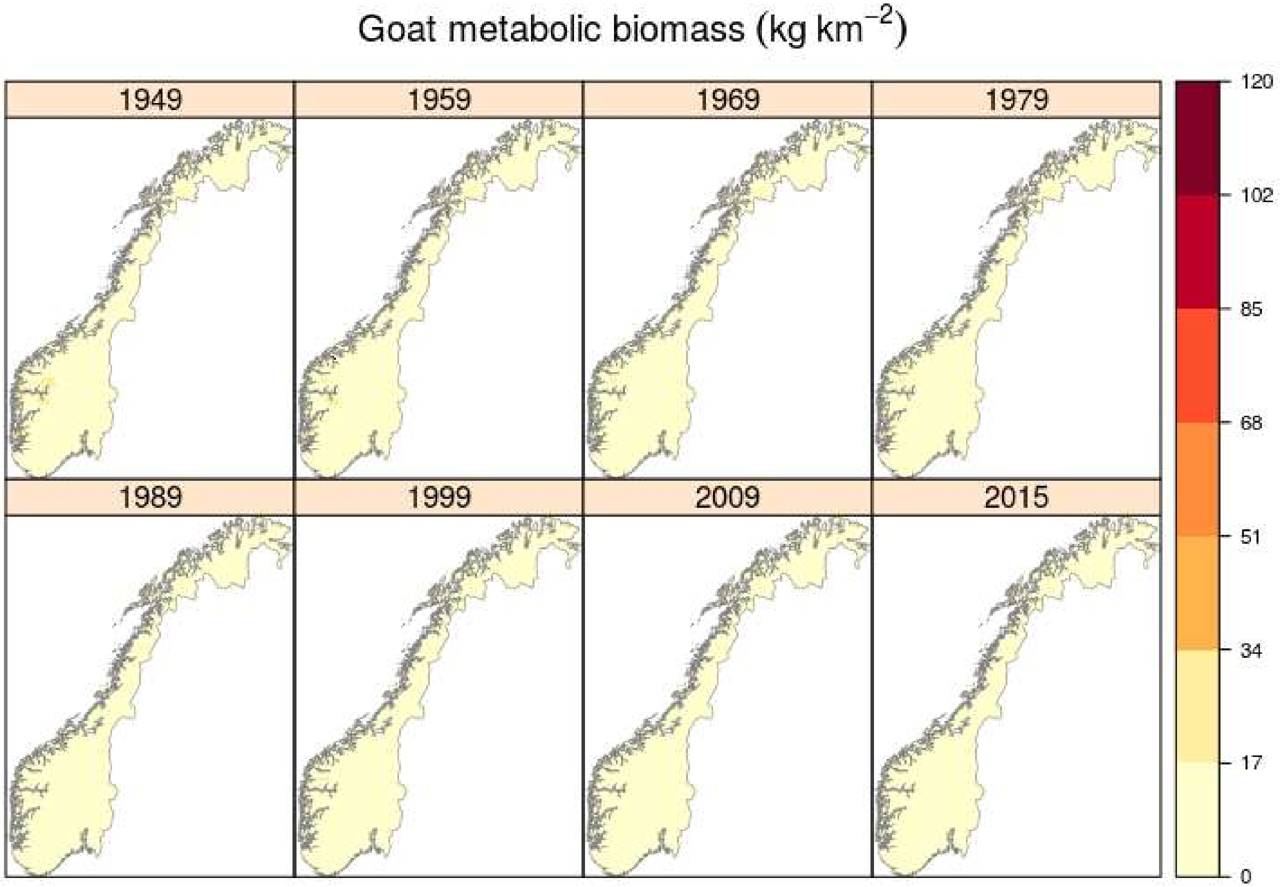

Fig P. Goat metabolic biomass (kg km^{-2}) across all years and municipalities in Norway.

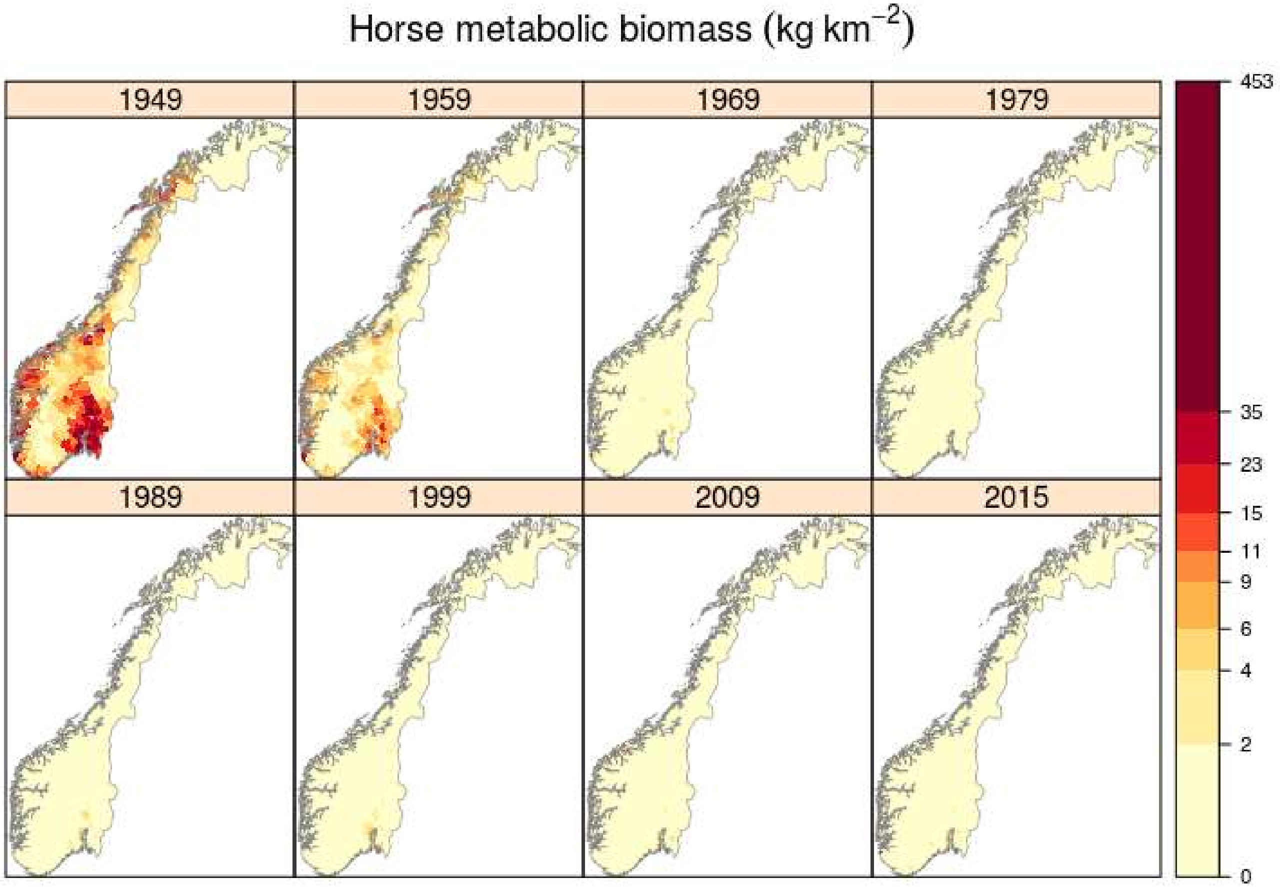

Fig Q. Horse metabolic biomass (kg km^{-2}) across all years and municipalities in Norway.

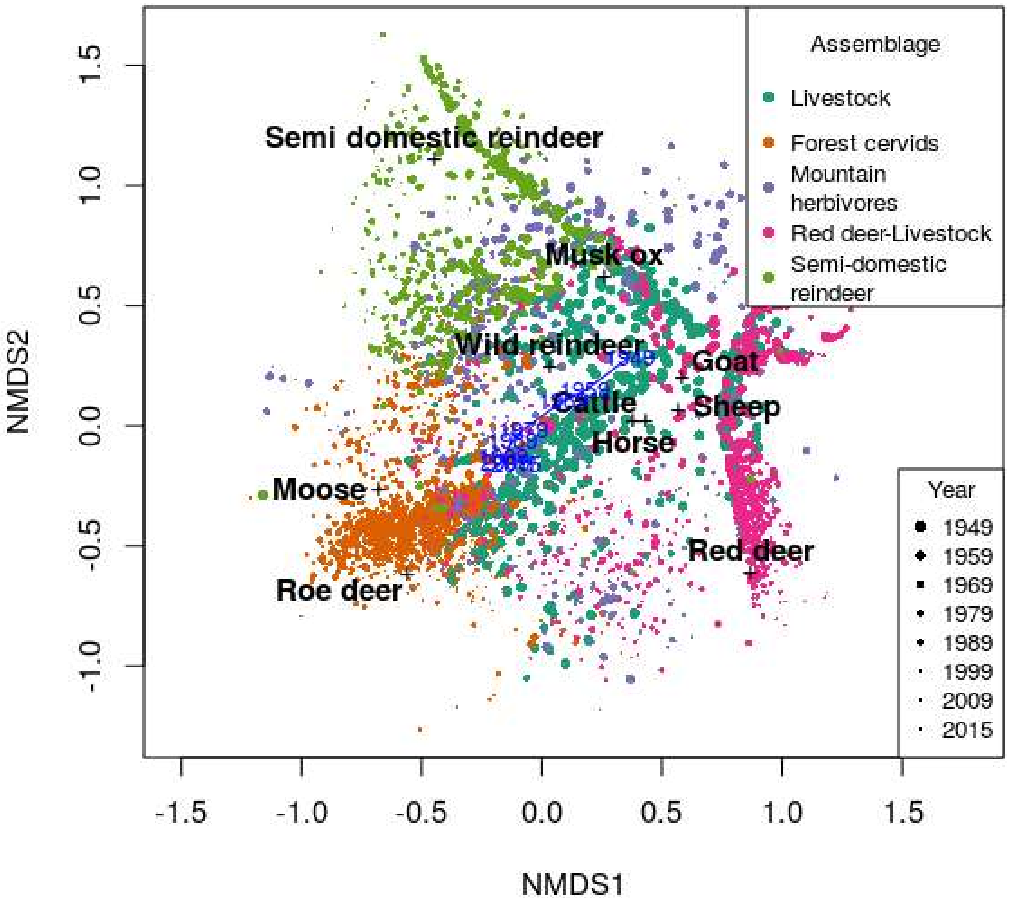

Fig R. Non-metric multidimensional scaling of large herbivores community composition in Norway between 1949 and 2015. Circular points represent municipalities with colour denoting the assemblage type and size the year with more recent periods smaller sized. Text and cross points show species scores. The blue line shows the trajectory of change in average herbivore assemblage over time (each year is labelled).

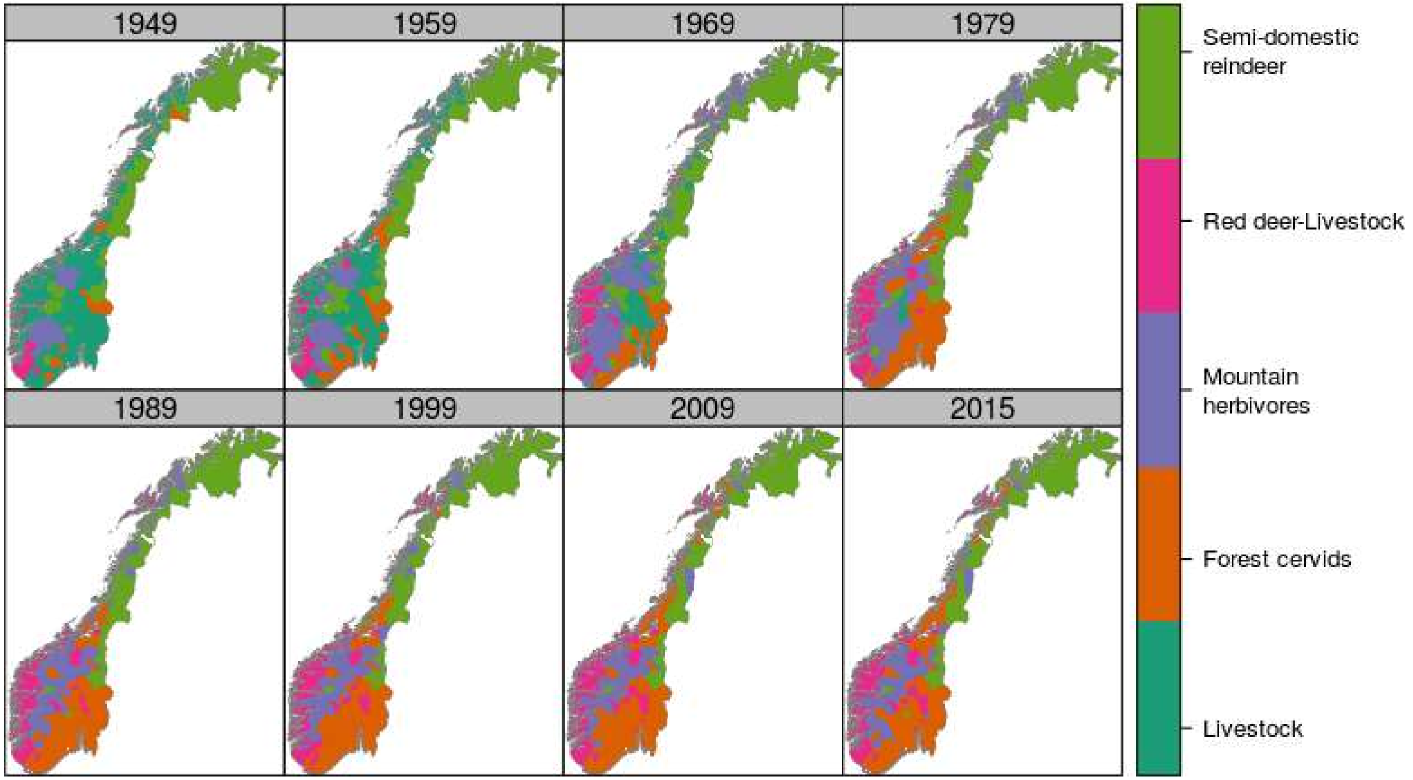

Fig S. The distribution of the five herbivore community clusters presented in Fig 4 across Norwegian municipalities and years. White shows municipalities with no herbivore data in 2015.

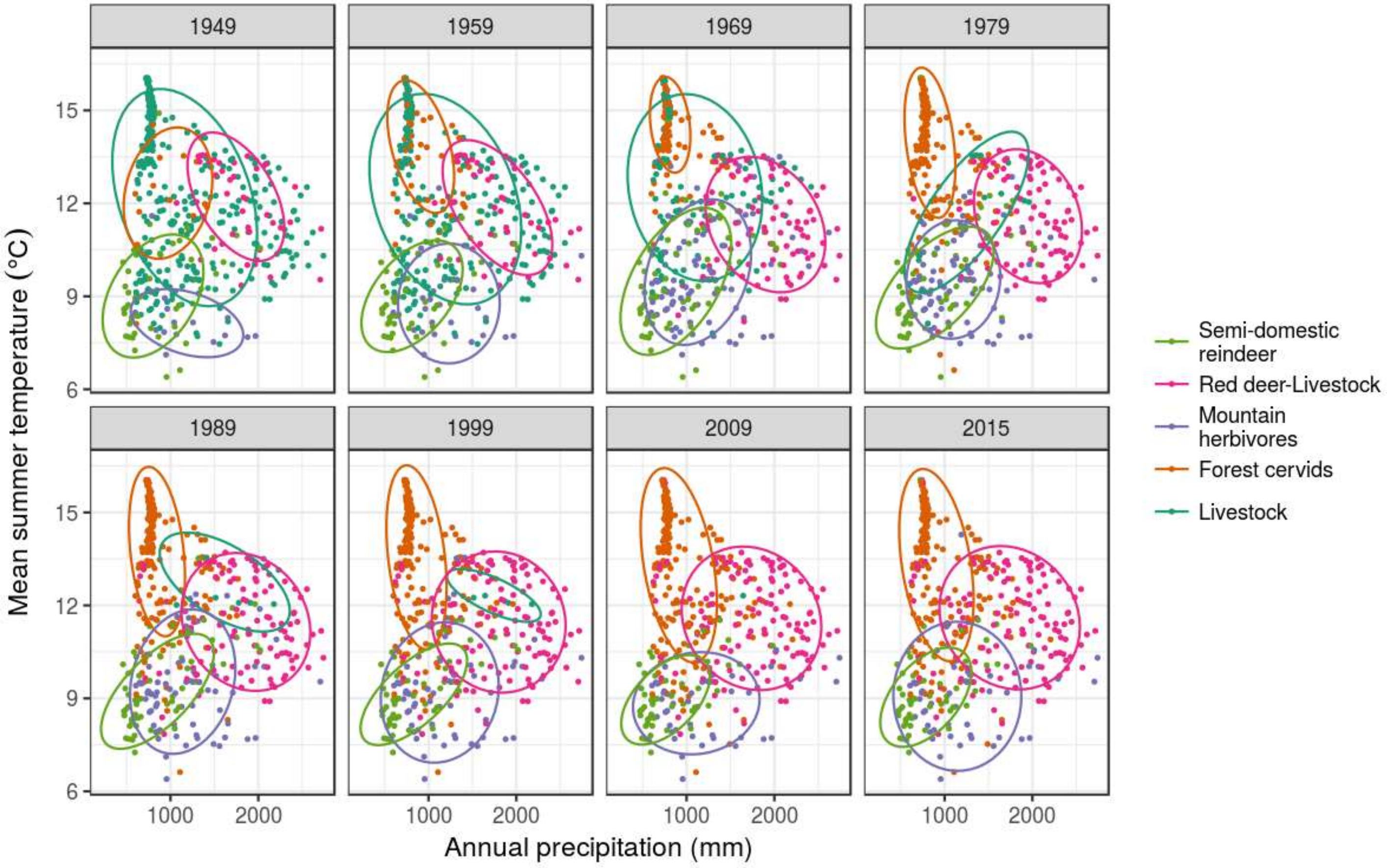

Fig T. Modified Whittaker plots showing the distribution of each municipality in terms of average annual precipitation and summer temperature (note that temperature increases up the y-axis). Points are coloured by the herbivore assemblage (Fig 4) characterising each municipality in each year. Ellipses show the 75% quartile of municipalities within each assemblage type. Note that the livestock assemblage is represented by only one municipality in 2009 and had disappeared altogether by 2015.
